# LYRUS: a machine learning model for predicting the pathogenicity of missense variants

**DOI:** 10.1093/bioadv/vbab045

**Published:** 2021-12-25

**Authors:** Jiaying Lai, Jordan Yang, Ece D Gamsiz Uzun, Brenda M Rubenstein, Indra Neil Sarkar

**Affiliations:** 1 Center for Biomedical Informatics, Brown University, Providence, RI 02903, USA; 2 Center for Computational Molecular Biology, Brown University, Providence, RI 02906, USA; 3 Department of Chemistry, Brown University, Providence, RI 02906, USA; 4 Department of Pathology and Laboratory Medicine, Brown University Alpert Medical School, Providence, RI 02903, USA; 5 Department of Pathology, Rhode Island Hospital, Providence, RI 02903, USA; 6 Rhode Island Quality Institute, Providence, RI 02908, USA

## Abstract

**Summary:**

Single amino acid variations (SAVs) are a primary contributor to variations in the human genome. Identifying pathogenic SAVs can provide insights to the genetic architecture of complex diseases. Most approaches for predicting the functional effects or pathogenicity of SAVs rely on either sequence or structural information. This study presents 〈Lai Yang Rubenstein Uzun Sarkar〉 (LYRUS), a machine learning method that uses an XGBoost classifier to predict the pathogenicity of SAVs. LYRUS incorporates five sequence-based, six structure-based and four dynamics-based features. Uniquely, LYRUS includes a newly proposed sequence co-evolution feature called the variation number. LYRUS was trained using a dataset that contains 4363 protein structures corresponding to 22 639 SAVs from the ClinVar database, and tested using the VariBench testing dataset. Performance analysis showed that LYRUS achieved comparable performance to current variant effect predictors. LYRUS’s performance was also benchmarked against six Deep Mutational Scanning datasets for PTEN and TP53.

**Availability and implementation:**

LYRUS is freely available and the source code can be found at https://github.com/jiaying2508/LYRUS.

**Supplementary information:**

Supplementary data are available at *Bioinformatics Advances* online.

## 1 Introduction

Recent technological advances, such as high-throughput screening methods, have made an abundance of sequencing data that have transformed our understanding of human genetic variation readily available. Since the determination of the first human genome sequence, more than one million human genomes have been collectively sequenced across the academic, clinical and private sectors (Fowler and Fields, 2014; Shendure *et al.*, 2017). This increase in genomic data is revealing a growing number of rare variants, for which there is insufficient data to decipher whether they are pathogenic. Rationalizing the functional and clinical implications of these millions of observed sequence variants remains a formidable undertaking.

In the post-genomic era, understanding the relationship among genetic and phenotypic variations represents a major challenge (Ormond *et al.*, 2010). A single amino acid variant (SAV) is an alteration in the protein sequence, which is a result of a missense single nucleotide variant (SNV). Among the known disease variants, roughly 45% are missense variants that encode a single amino acid change in the affected protein (Marinko *et al.*, 2019), which are tied to human diseases, such as Parkinson’s disease, Alzheimer’s disease and cancer (Niu *et al.*, 2016; Yip *et al.*, 2008). Differentiating pathogenic SAVs from neutral SAVs is thus of great importance in the post-genomic era, as it can enhance our understanding of the correlation between genotype and phenotype, facilitating the development of novel treatment strategies for complex diseases.

The accurate classification of effects of genetic variants on various disorders remains a difficult goal to achieve, despite the abundance of genomic data collected over the last decade and the multiple efforts to elucidate their links to phenotypic traits. Most existing software for predicting the functional effects of amino acid variations are based on the assumption that protein sequences observed among living organisms have survived natural selection. As a result, evolutionarily conserved amino acid positions across multiple species are assumed to be functionally important, and amino acid variations observed at conserved positions are assumed to be pathogenic (Yue *et al.*, 2005).

Previous analyses have shown that methods incorporating only sequence-related information may suffer from reduced accuracy (Saunders and Baker, 2002). Furthermore, Sunyaev *et al.* (2000) have shown that pathogenic mutations often affect the intrinsic structural features of proteins, including sites involved in disulfide bonds. Wang and Moult (2001) have demonstrated that most pathogenic mutations appear to affect protein stability. It is therefore evident that knowing the impact of mutations on protein stability is essential for clarifying the relationships among the structure, function and dynamics of a given protein. Structure-based modeling approaches have lagged behind sequence-based approaches in evaluating the effects of SAVs, even though first-generation classifiers that can take 3D structures into account have shown considerable success (Adzhubei *et al.*, 2010; Ancien *et al.*, 2018; Capriotti and Altman, 2011). Additionally, most computational methods focus on reaching the highest variant classification accuracy rather than understanding the modifications that occur at the molecular scale, which might be crucial for the design of drugs or treatments.

Changes in folding free energies (ΔΔ*G*_fold)_ are the standard thermodynamic measures to probe the effects of mutations on protein stability and have already been demonstrated to characterize sequence and structural patterns among human pathogenic amino acid variants (Joerger and Fersht, 2007; Peng and Alexov, 2016; Petukh *et al.*, 2015; Yang *et al.*, 2020). Several computational approaches have been developed to predict ΔΔ*G*_fold_ as a means to link it to the pathogenicity of mutations (Blanco *et al.*, 2018; Cang and Wei, 2017; Getov *et al.*, 2016; Li *et al.*, 2014; Zhang *et al.*, 2012). Besides changes in folding free energies, solvent accessibility has been known to be associated with the pathogenicity of SAVs. SAVs located on the protein surface are more likely to be neutral, whereas those buried in the protein core are more likely to be pathogenic (Yue *et al.*, 2005). Accordingly, various approaches for predicting pathogenicity that rely on structural features are available, such as Bongo, which uses graph theoretic measures to evaluate the structural impacts of single point mutations (Bao and Cui, 2005; Capriotti and Altman, 2011; Cheng *et al.*, 2008). Other studies have shown that structural information can provide results of comparable quality to those that use sequence and evolutionary information in predicting pathogenic SAVs (Jacobs *et al.*, 2001; Kannan and Vishveshwara, 1999; Vendruscolo *et al.*, 2002).

In addition to sequence conservation and protein structure, protein dynamics have also been proven to be useful for predicting SAV functional impacts. Ponzoni and Bahar (2018) evaluated a set of features generated by elastic network models of proteins to efficiently screen protein dynamics. Their study shows the utility of considering the equilibrium dynamics of the protein as a means of improving the predictive ability of current pathogenicity predictors. Other dynamic features, such as stiffness, effectiveness and sensitivity, have also been shown to be important in pathogenicity prediction (Smith *et al.*, 2019). Tools that use dynamics-based features (e.g. Rhapsody) demonstrate that predictions are improved when dynamics-based and sequence-based features are combined (Ponzoni *et al.*, 2020).

Picking the most suitable machine learning (ML) algorithm that can learn the most salient of these many possible features for prediction can be challenging. The Tree-based Pipeline Optimization Tool (TPOT) is an evolutionary algorithm-based automated machine learning (autoML) system that uses genetic programming to optimize a series of feature selectors, pre-processors and ML models to maximize classification/regression accuracy and recommend an optimal pipeline (Banzhaf *et al.*, 1998). TPOT has been shown to frequently outperform standard ML analyses given no *a priori* knowledge about the problem. We utilized TPOT to search for the best ML pipeline for our dataset.

We introduce LYRUS, an ML-based approach that incorporates the essential properties of structural information, evolutionary conservation and protein dynamics, to predict the pathogenicity of SAVs. We included a recently developed sequence-evolutionary-based concept, called variation number, which has been shown to vary significantly among pathogenic and neutral variants in BRCA1 and BRCA2 SNVs (Lai and Sarkar, 2020). The inclusion of variation number distinguishes LYRUS from tools currently used in the field. LYRUS was trained on a large set of human protein variations obtained from ClinVar, which is a publicly accessible database (Landrum *et al.*, 2018). The performance of LYRUS was assessed on the ClinVar training dataset as well as an independent VariBench dataset (Nair and Vihinen, 2013), and compared to that of PolyPhen2, PROVEAN, SIFT, Rhapsody, EVMutation, MutationAssessor, SuSPect, FATHMM, MVP, PrimateAI, UNEECON, M-CAP and REVEL (Adzhubei *et al.*, 2010; Choi *et al.*, 2012; Hopf *et al.*, 2017; Huang, 2020; Ioannidis *et al.*, 2016; Jagadeesh *et al.*, 2016; Kumar *et al.*, 2009; Ponzoni *et al.*, 2020; Qi *et al.*, 2021a; Reva *et al.*, 2011; Shihab *et al.*, 2013; Sundaram *et al.*, 2018; Yates *et al.*, 2014). To truly exam the predictive power of LYRUS in an unbiased fashion, we also performed an independent assessment by benchmarking LYRUS and other variant effect predictors (VEPs) against three phosphatase and tensin homolog deleted on chromosome 10 (PTEN) and three tumor protein 53 (TP53) Deep Mutational Scanning (DMS) datasets.

## 2 Methods

### 2.1 Training dataset

The training dataset for the ML pipeline was generated using ClinVar (Landrum *et al.*, 2018). Each entry in ClinVar is associated with a review score: the larger the number of review stars an entry receives up to a maximum of four, the more verified that entry has been. All of the SAVs in ClinVar with at least one review star were obtained. The SAVs in the resulting dataset were further categorized as having a pathogenicity of benign, benign/likely benign, likely benign, likely pathogenic, pathogenic/likely pathogenic or pathogenic. Benign, benign/likely benign and likely benign SAVs were assigned a pathogenicity score of 0, while all other SAVs were assigned a score of 1. The number of ClinVar SAVs is listed in Supplementary Table S1 and Figure S1.

### 2.2 Feature selection for SAV pathogenicity prediction

The three categories of features widely used in SAV pathogenicity prediction are sequence-based features, structure-based features and dynamics-based features. We picked 15 features in total from these three categories in our prediction pipeline (Table 1 and Supplementary Table S2). The variation number is a recently developed phylogenetic measure that quantifies sequence conservation using sequence orthologs from different species (Lai and Sarkar, 2020). The pipeline for calculating variation numbers is depicted in Supplementary Figure S2. The orthologous sequences required by the variation number and EVMutation were obtained from the NCBI Orthologs Database (accessed in October, 2020) (NCBI, 2018). In addition to the sequence and variant information, all of the structural and dynamic features also require protein structure files from the Protein Data Bank (PDB). The PDB files were downloaded from SWISS-MODEL (Kiefer *et al.*, 2009).

**Table 1.** Features used for SAV pathogenicity prediction

Feature name	Description	Type
Variation number	Sequence position conservation score calculated using orthologs	SEQ
	Variation numbers employed in the model are scaled using min. to max. normalization for each amino acid sequence	
Δ*E* epistatic score	Change in evolutionary statistical energy computed by EVmutation (Hopf *et al.*, 2017)	SEQ
Functional impact score (FIS)	Predicted magnitude of the effects of amino acid substitutions	SEQ
	weighted by the relative frequency of disease-causing and neutral	
	amino acid substitutions computed by FATHMM (Shihab *et al.*, 2013)	
ΔPSIC	Difference of PSIC scores for two amino acid residue variants	SEQ
	computed by PolyPhen-2 (Adzhubei *et al.*, 2010)	
Wild-type PSIC	PSIC score for wild-type amino acid residue computed by PolyPhen-2 (Adzhubei *et al.*, 2010)	SEQ
ΔΔ *G*_fold_	Folding free energy difference computed by FoldX (Schymkowitz *et al.*, 2005)	STR
SASA	Solvent accessible surface area computed by FreeSASA (Mitternacht, 2016)	STR
Mutant SSF	Knowledge-based potential for mutant amino acid variants	STR
	computed by MAESTRO (Laimer *et al.*, 2015)	
Active site value	Calibrated probability of being a ligand-binding residue	STR
	Assigned 1 if the probability is >0.5	
	computed by P2Rank (Krivák and Hoksza, 2018)	
Mutant reference energy	Unfolded-state reference energies for mutant amino acid variants	STR
	computed by PyRosetta (Alford *et al.*, 2017)	
ΔReference energy	Difference between unfolded-state reference energies for two amino acid variants	STR
	computed by PyRosetta (Alford *et al.*, 2017)	
MSD	Mean squared displacements of $C_{\alpha }$ atoms derived from the anisotropic network model	DYN
	computed by *ProDy* (Bakan *et al.*, 2011)	
Mechanical stiffness	Measurement of the mechanical resistance of residues to external pulling forces	DYN
	computed by *ProDy* (Bakan *et al.*, 2011)	
Effectiveness	The ability of a residue to transmit mechanical deformation signals	DYN
	when subjected to a unit perturbation computed by *ProDy* (General *et al.*, 2014)	
Sensitivity	The ability of a residue to sense mechanical deformation signals	DYN
	when subjected to a unit perturbation computed by *ProDy* (General *et al.*, 2014)	

*Note*: Fifteen features belonging to three different categories are used. Each feature calculation requires either an amino acid sequence or PDB file, or both. SEQ, sequence-based feature; STR, structure-based feature; DYN, dynamics-based feature.

Principal component analysis (PCA) is a way of identifying patterns in data that highlights their similarities and differences. The target dataset can be compressed by performing a PCA that reduces its number of dimensions if the data’s cumulative variance does not drop below a desired threshold, i.e. if there is not too much loss of information. Redundancy was analyzed for the 15 selected model features using PCA.

### 2.3 ML model selection and evaluation

TPOT was used in this study to determine the ML pipeline with the highest accuracy for our training dataset (Le *et al.*, 2020). About 80% of the dataset was used for training and 20% was used for testing. For TPOT parameters, both the number of generations and population size were set to 100, the cross-validation size was set to 5 and the verbosity was set to 2. TPOT suggested an XGBoost classifier to be the most suitable for our training dataset. The XGBoost algorithm, originally created by Chen and Guestrin, is a scalable tree boosting system that has been widely used by researchers (Chen and Guestrin, 2016).

Ten-fold cross-validation was performed to assess the performance of LYRUS using the ClinVar dataset. The accuracy, sensitivity, specificity, *F*-measure and Matthews correlation coefficient (MCC) were calculated, as defined below:

$$\begin{array}{c} A\mathrm{ccuracy}=\frac{TP+TN}{TP+TN+FP+FN}\\ S\mathrm{ensitivity}=\frac{TP}{TP+FN}\\ S\mathrm{pecificity}=\frac{TN}{TN+FP}\\ F_{1}=\frac{TP}{TP+\frac{1}{2}(FP+FN)}\\ MCC=\frac{TP\times TN-FP\times FN}{\sqrt{\left(TP+FN)\times (TP+FP)\times (TN+FN)\times (TN+FP\right)}}. \end{array}$$

The performance of the chosen ML pipeline was compared to that of PolyPhen-2 (Adzhubei *et al.*, 2010), PROVEAN (Choi *et al.*, 2012), SIFT (Kumar *et al.*, 2009), Rhapsody (Ponzoni *et al.*, 2020), EVMutation (Hopf *et al.*, 2017), MutationAssessor (Reva *et al.*, 2011), SuSPect (Yates *et al.*, 2014), FATHMM (Shihab *et al.*, 2013), MVP (Qi *et al.*, 2021a), PrimateAI (Sundaram *et al.*, 2018), UNEECON (Huang, 2020), M-CAP (Jagadeesh *et al.*, 2016) and REVEL (Ioannidis *et al.*, 2016). PolyPhen-2, PROVEAN, SIFT, Rhapsody, EVMutation, MutationAssessor, SuSPect, FATHMM and MVP were accessed in October 2020, and PrimateAI, UNEECON, M-CAP and REVEL were accessed in June 2021 for the ClinVar dataset. The source of each method is available in Supplementary Table S3 and the thresholds for classifying pathogenic and benign SAVs used by each method are available in Supplementary Table S4. Not all VEPs were able to predict all of the SAVs in ClinVar, thus the missing predictions (if there were any) were imputed for each VEP to benefit VEPs with few missing values.

In addition to the ClinVar dataset, the performance of LYRUS was also evaluated against other VEPs using the VariBench testing dataset (Supplementary Table S1, accessed in July, 2021) (Nair and Vihinen, 2013). Two datasets from VariBench were obtained for performance testing, VariBench_selected and VariBench_limited. The VariBench_selected dataset contains all SAVs whose protein structure is not present in the ClinVar dataset. This was done to prevent the Type I and II data circularity issues described in Grimm *et al.* (2015). The VariBench_limited dataset is selected from the VariBench_selected dataset, and further filtered such that all the VEP predictions are available, except for those from EVMutation. EVMutation predictions were excluded from the VariBench_limited dataset because too many predictions were missing. Rhapsody was excluded from both VariBench datasets because Rhapsody was trained using the VariBench dataset. All of the VEPs were accessed as of July 2021.

### 2.4 DMS dataset selection for correlation analysis

PTEN and TP53 are noteworthy proteins as they both have a number of different DMS datasets. We pulled three PTEN DMS datasets from two different studies and three TP53 datasets from one study; namely, pten(a) (Matreyek *et al.*, 2018), pten(b) (Mighell *et al.*, 2018), pten(highqual_b) (Mighell *et al.*, 2018), p53(wt_nutlin) (Giacomelli *et al.*, 2018), p53(null_nutlin) (Giacomelli *et al.*, 2018) and p53(null_etoposide) (Giacomelli *et al.*, 2018). The screen for the pten(b) dataset assessed the disruption of an artificial gene circuit in yeast, which probed phosphatase activity. The pten(highqual_b) dataset was created by the same group, which obtained high confidence data based on low standard error or replicate concordance. The phenotypic screen for pten(a) measured protein abundance in the cell by fluorescence of EGFP bound to the protein. We also selected three TP53 datasets from one study done by Giacromelli *et al.* p53(wt_nutlin) dataset was generated by first creating isogenic WT *TP53* A549 human lung carcinoma cell populations using CRISPR-Cas9-mediated gene editing. Then, the differential responses of these isogenic cells were capitalized to a p53-activating agent, nutlin-3 and pooled positive selection screens were subsequently performed. p53(null_nutlin) dataset was generated by creating isogenic null *TP53* A549 human lung carcinoma cell populations using CRISPR-Cas9-mediated gene editing. Then, the differential responses of isogenic cells were capitalized to the same p53-activating agent, nutlin-3 and pooled positive selection screens were performed. p53(null_etoposide) dataset differs from p53(null_nutlin) in that the responses of those isogenic cells were capitalized to another p53-activating agent, etoposide (Giacomelli *et al.*, 2018).

## 3 Results

### 3.1 Feature validation

**Fig. 1.**
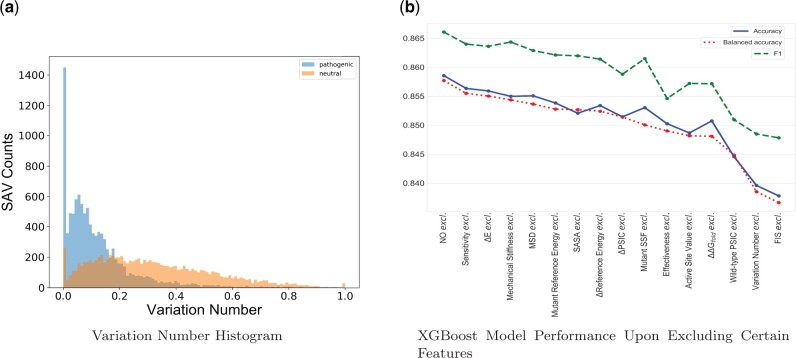
Feature validations. (**a**) A comparison of variation number histograms for the pathogenic and neutral SAVs. Among the 22 639 selected SAVs, 9743 SAVs were neutral and 10 564 SAVs were pathogenic. The mean variation number for the pathogenic SAVs was 0.12 while the mean variation number for the neutral SAVs was 0.32. (**b**) The XGBoost model was trained 15 times, each time excluding one feature from training. The results are shown with the accuracy, balanced accuracy and *F*1 scores calculated using the best model plotted on the *y*-axis

Figure 1a shows the histograms of variation numbers for the pathogenic and neutral SAVs. Variation numbers range from 0 to 1, where 0 means high conservation and 1 means low conservation. The mean variation numbers for the pathogenic SAVs is 0.12, while the mean variation number for the neutral SAVs is 0.32. The variance of the variation numbers for the pathogenic SAVs is 0.017, and the variance of the variation numbers for the neutral SAVs is 0.04. A *t*-test was performed using variation numbers for pathogenic and neutral SAVs (Virtanen *et al.*, 2020). The resulting *t*-statistic is −80.33, with a *P*-value of 0.0. The *t*-test results suggest that variation numbers for pathogenic and neutral SAVs are significantly different: pathogenic SAVs have smaller variation numbers than neutral SAVs, which suggests that pathogenic SAVs are more conserved than neutral SAVs.

The calculation of variation number for each amino acid sequence depends on its orthologous sequences. Different amino acid sequences do not have equal numbers of orthologous sequences. To test whether the power of our model is affected by the number of orthologous sequences, we did a stratifying test by training and testing the model accuracy based on the number of orthologous sequences. We were able to download the orthologous sequences for 4354 proteins through the NCBI Orthologs database (NCBI, 2018). Among all of the proteins, 95%, 92%, 86%, 79%, 56% and 42% have at least 50, 100, 150, 200, 250 and 300 orthologous sequences, correspondingly. We trained our model using SAVs with at least 50, 100, 150, 200, 250 or 300 orthologous sequences. We then applied each model to the whole dataset and calculated the accuracy using 10-fold cross-validation. The accuracies for each model were similar, as shown in Supplementary Figure S3. Furthermore, more than 95% of the proteins have more than 50 orthologs, so the impact of proteins with <50 orthologs would be minimal. Thus, the number of orthologs used to compute the variation number had minimal impact on our model.

PCA was applied to our feature dataset with the objective of cross-validating our feature selections and checking redundancy among our 15 features. Supplementary Figure S4 shows the correlation between the cumulative variance (i.e. the sum of the variances of the individual principal components) and the number of principal components. The plot shows that 12 components are needed to describe 90% of the variance in the calculated results of all SAVs’ 15 features. Because most of the population variance cannot be attributed to the first few components, they cannot replace the original variables without loss of information. This analysis validates that there is minimal redundancy in our dataset and further supports the use of the selected features in the subsequently chosen ML model.

In addition to the PCA, Pearson correlations were calculated between all pairs of features, as depicted in Supplementary Figure S5. The top features that have the highest correlation with clinical scores are the wild-type PSIC, Δ PSIC, functional impact score and variation number, which are all sequence-based features. Four pairs of features have a (negative) correlation >0.5. The wild-type PSIC and ΔPSIC have a correlation of 0.66, the wild-type PSIC and variation number have a negative correlation of −0.59, the solvent accessible surface area (SASA) and mechanical stiffness have a negative correlation of −0.53 and the mutant reference energy and mutant statistical scoring function (SSF) have a negative correlation of −0.52. All other pairs of features have (negative) correlations smaller than 0.5. The correlation heatmap of the raw data suggests that sequence features have a larger correlation with pathogenicity than the structural and dynamic features. It also shows that all of the features are largely independent of one another, and thus the inclusion of all of the features in our model is necessary.

To illustrate the necessity of incorporating the variation number into our model, we trained our model 15 times, each time excluding one feature from training. After training 15 times, we obtained 15 models, and each model corresponds to the best predictive model with one specific feature excluded. We subsequently calculated the corresponding accuracy, balanced accuracy and *F*1 scores associated with each model, and the results are shown in Figure 1b. When variation number was excluded from the predictive pipeline, accuracy, balanced accuracy and *F*1 score dropped ∼3%, the second-biggest drop observed when compared with the drops that accompanied excluding other features. Thus, the inclusion of variation number as a feature is necessary.

### 3.2 ML pipeline

A classification model is intended for predicting whether an SAV is pathogenic (Score 1) or non-pathogenic (Score 0). TPOT recommended the XGBoost Classifier, which achieved the highest accuracy of 0.859, as the most suitable ML method for our dataset (Chen and Guestrin, 2016; Le *et al.*, 2020). The optimized XGBoost classifier has a learning rate of 0.1. Feature importance scores (Supplementary Fig. S6) showed that ΔPSIC has the highest weight, followed by FIS, wild-type PSIC and variation number, which are all sequence-based features. This is all in accordance with the feature correlation heatmap (Supplementary Fig. S5). All of the other features had smaller, but similar importance values.

### 3.3 Predictive power of the model

**Fig. 2.**
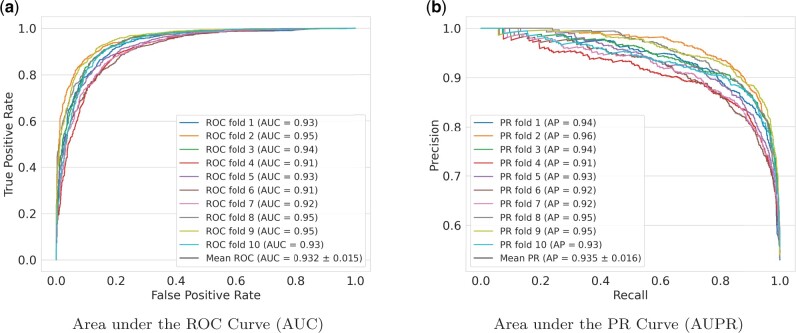
Comparison of the ROC and PR curves of LYRUS using 10-fold cross-validation with the ClinVar dataset. (**a**) ROC curves for each fold of the 10-fold cross-validation, the mean and the standard deviation. (**b**) PR curves for each fold of the 10-fold cross-validation, the mean and the standard deviation

A total of 22 639 SAVs were extracted from ClinVar. The performance of LYRUS was tested using 10-fold cross-validation with the ClinVar dataset. Figure 2 shows the receiver operating characteristic (ROC) curve and the precision–recall (PR) curve for the 10-fold cross-validation for LYRUS. The mean area under the receiver operating characteristic (AUROC) is 0.932 and the mean area under the precision–recall (AUPR) is 0.935. We also plotted both the ROC and PR curves for the other 13 VEPs (Supplementary Fig. S7). REVEL had a slightly higher AUROC of 0.937 than LYRUS. REVEL, M-CAP and Rhapsody also had a higher AUPR than LYRUS. Supplementary Table S5 and Figure S8 present the accuracy, sensitivity, specificity, *F*-measure (*F*_1_) and MCC for the 14 VEPs. LYRUS achieved the second highest accuracy, specificity, *F*-measure and MCC. The sensitivity of LYRUS is lower than that of PolyPhen2, PROVEAN, SIFT, MVP, M-CAP and REVEL. Statistical analysis demonstrates that LYRUS performs comparably to other VEPs.

**Fig. 3.**
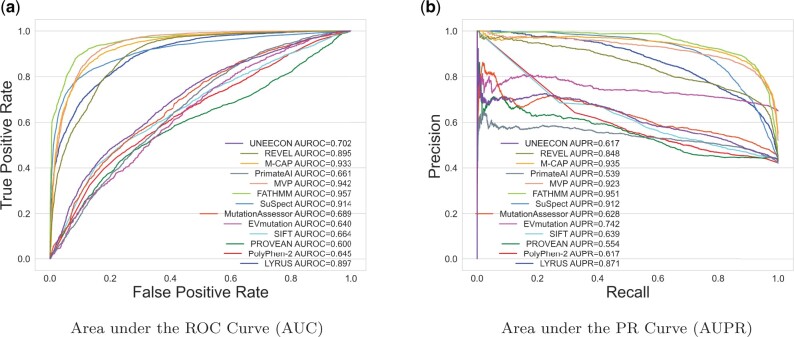
Comparison of ROC and PR curves of LYRUS and 12 other VEPs using VariBench_selected dataset. (**a**) ROC curves for the 13 VEPs. LYRUS has an AUROC of 0.891, which is the fifth highest among all the VEPs. (**b**) PR curves for the 13 VEPs

In addition to the ClinVar dataset, performance analysis was also done using the VariBench dataset. The VariBench_selected dataset contains 8223 SAVs, which includes 3466 pathogenic SAVs (42%) (Supplementary Table S1). Figure 3a shows the ROC curve, and Figure 3b shows the PR curve for the VariBench_selected dataset. LYRUS has an AUROC of 0.897, and an AUPR of 0.871. M-CAP, MVP, FATHMM and SuSPect have higher AUROC and AUPR than LYRUS. The accuracy, sensitivity, specificity, *F*-measure (*F*_1_) and MCC are shown in Supplementary Table S6 and Figure S9. LYRUS had lower accuracy than SuSPect, FATHMM and MVP. The performance of LYRUS on the VariBench_limited dataset was similar to that of the VariBench_selected dataset (Supplementary Figs S10 and S11 and Table S7). Although LYRUS did not achieve the best performance among all VEPs, LYRUS’s performance is close to that of other VEPs in the field. LYRUS achieved lower accuracy than SusPect, FATHMM, MVP and M-CAP. However, it is worth noting that MVP and M-CAP are meta-predictors (Jagadeesh *et al.*, 2016; Qi *et al.*, 2021b). SuSPect included 77 features (Yates *et al.*, 2014), which is far more than the 15 features incorporated in LYRUS. Furthermore, we did not exclude those SAVs that have been used as training data in the other VEPs from both the ClinVar and VariBench datasets, thus all the other VEPs might have an advantage over LYRUS by having overlapping SAVs between the training and testing dataset.

### 3.4 Illustrative applications

To illustrate the effectiveness of LYRUS for identifying pathogenicity from neutral variants, we present a case study of two proteins, PTEN and tumor protein 53 (TP53). Before being applied to PTEN and TP53, LYRUS was retrained on datasets that excluded the SAVs of these two proteins.

### 3.5 Pathogenicity of PTEN mutants

PTEN is associated with advanced-stage or metastatic cancers (Li *et al.*, 1997; Liaw *et al.*, 1997; Li and Sun, 1997). LYRUS was applied to a dataset of 7657 (403×19) SAVs of PTEN. PTEN (UNIPROT: P60484) has 403 amino acids. However, the complete X-ray crystal structure for PTEN is unavailable. The PDB 1D5R structure was used as a template to simulate PTEN using the Robetta server (Song *et al.*, 2013). Simulated structures were used for the PTEN amino acids 1–13, 282–312 and 352–403, which are missing from the crystal structure. The prediction results for PTEN are shown in Supplementary Figure S12. Most SAVs in PTEN are predicted to be pathogenic, but all possible 342 mutants from Thr286 to Ser305 except Asn292 and Gly293 were predicted to be neutral. These positions are all located on the surface of the protein (Supplementary Fig. S13a), and the neutral predictions are due to their low ΔPSIC scores, negative or small positive ΔΔ*G*_fold_ values, large SASA values, low stiffness values and large MSD values.

There are 110 PTEN SAVs with a ‘review star’ of at least one in ClinVar (Landrum *et al.*, 2018). The 14 VEPs were evaluated on these 110 SAVs. The results are listed in Supplementary Table S8 and Figure S14. All the VEPs have similar performance, which may be caused by an imbalance of the dataset, as 108 out of 110 SAVs are pathogenic. We further examine the SAVs whose pathogenicity is incorrectly predicted by LYRUS. There are four false negative SAVs: R15K, Y16H, P246L and R335Q (Supplementary Fig. S13b). R15K is predicted to be neutral given its low ΔPSIC and WT PSIC values. ΔPSIC scores indicate the difference between the profile scores [obtained from computing the profile matrix (Sunyaev *et al.*, 1999)] of the two allelic variants in the polymorphic position (Adzhubei *et al.*, 2010). Large positive values of this difference suggest that the studied substitution is rarely or never observed in the protein family. R15K’s small positive ΔPSIC value implies that this specific substitution is frequently observed in the protein family and hence less likely to be pathogenic (Sunyaev *et al.*, 1999). The same rationale can be used to explain the remaining three false negative predictions (i.e. Y16H, P246L and R335Q) even though their positive ΔΔ*G*_fold_ values would suggest that they are destabilizing mutations. Interestingly, variation numbers of all four SAVs are relatively low, indicating that these four sites are highly conserved. This finding also demonstrates the efficacy of variation number in pathogenicity prediction.

### 3.6 Pathogenicity of TP53 mutants

LYRUS was also applied to TP53, which encodes a multifunction transcription factor whose loss promotes tumor formation (Vousden and Lu, 2002). The predicted probabilities of pathogenicity of the TP53 variants are presented in Supplementary Figure S15. The region spanning codons 100–290 is predicted to be highly pathogenic. This region contains the core domain of the TP53 protein, and mutations in the core domain can result in the loss of DNA binding activity (Cho *et al.*, 1994). In addition, more than 80% of somatic TP53 mutations in human cancers occur in this region (Cho *et al.*, 1994; Olivier *et al.*, 2010). These findings validate our predictions. The performance of LYRUS was also compared with that of 13 other VEPs using 142 ClinVar entries (Supplementary Table S9 and Fig. S16). LYRUS’s performance is comparable to other VEPs. Furthermore, FATHMM, MVP and M-CAP achieved a sensitivity of 1.0 but also a specificity of 0, which can be a concern. There are six false positive predictions by LYRUS: N263S, Y107H, R235S, R110H, G293W and H296Y. These SAVs are located on the surface of the protein and are hence solvent-exposed (Supplementary Fig. S17a). They are all predicted to be pathogenic due to their high ΔPSIC and SASA values and low Δ*E* and FIS values. Additionally, R337H, a variant located on an *α*-helix, was falsely predicted to be benign (Supplementary Fig. S17b).

### 3.7 Assessment of VEPs using DMS data

**Fig. 4.**
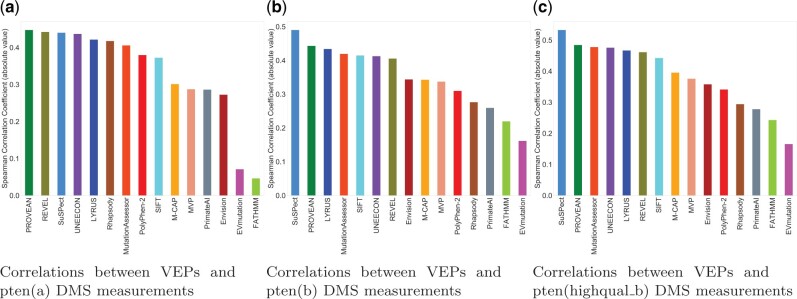
VEPs benchmarked against three PTEN DMS datasets (**a**) Spearman’s correlation (absolute value) between pten(a) DMS results, and 15 VEPs. The top three performing predictors are: PROVEAN, REVEL and SuSPect. (**b**) Spearman’s correlation (absolute value) between pten(b) DMS results, and 15 VEPs. The top three performing predictors are: SuSPect, PROVEAN and LYRUS. (**c**) Spearman’s correlation (absolute value) between pten(highqual_b) DMS results, and 15 VEPs variants. The top three performing predictors are: SuSPect, PROVEAN and MutationAssessor

**Fig. 5.**
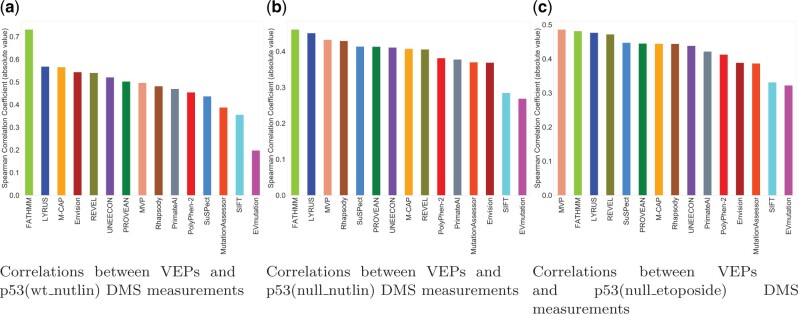
VEPs benchmarked against three TP53 DMS datasets (**a**) Spearman’s correlation (absolute value) between p53(wt_nutlin) DMS results and 12 VEPs. The top three performing predictors are: FATHMM, LYRUS and M-CAP. (**b**) Spearman’s correlation (absolute value) between p53(null_nutlin) DMS results and 12 VEPs. The top three performing predictors are: FATHMM, LYRUS and MVP. (**c**) Spearman’s correlation (absolute value) between p53(null_etoposide) DMS results and 12 VEPs variants. The top three performing predictors are: MVP, FATHMM and LYRUS

Missense variant databases curated from the literature rely on manual curation for data extraction and entry; the curation process of VariBench could also introduce systematic biases (Qi *et al.*, 2021b). To assess the performance of LYRUS in an unbiased fashion, we evaluated its predictive power on six independent DMS datasets that were not used for the development of LYRUS (Supplementary Table S10). Benchmarking DMS datasets to serve as performance estimators is backed up by two rationales: first, DMS experiments yield large-scale datasets that can directly reveal damaging mutations, and second, these datasets are entirely independent of any training and testing data used by the VEPs [except Envision (Gray *et al.*, 2018)]. Some VEPs failed to generate prediction results for some proteins or some SAVs. This can occur due to insufficiently deep multiple sequence alignments, mapping errors, lacking experimental PDB structural files or insufficient structural file coverage. To obtain a measure of relative performance for each predictor, we calculated Spearman’s correlation coefficient between independent DMS scores for PTEN and TP53 and the predictions of LYRUS and 14 other VEPs (Figs 4 and 5). LYRUS was the overall top-performing method for predicting PTEN and TP53 DMS results, showing the overall highest correlations out of all VEPs. It ranked within the top five predictors for all three PTEN DMS datasets and within the top three for all three TP53 DMS datasets. In addition, it consistently exhibited a strong correlation with every DMS dataset (Supplementary Fig. S18). Mighell *et al.* provided both their full fitness scores [pten(b)] and a filter for high-quality results [pten(highqual_b)] for PTEN (Mighell *et al.*, 2018). In cases with lower- and higher-quality data, we found that the filtered high-quality results have a slightly higher average correlation with the VEPs (Fig. 4b and c).

Of all VEPs, FATHMM produced the most divergent results, generating predictions with by far the highest correlation with TP53 DMS datasets but having unexpectedly low correlations for PTEN (Figs 4 and 5). This may result from overfitting the predictor to specific proteins, given the enrichment of TP53 mutations in the human disease databases (Shihab *et al.*, 2013) compared to other proteins in the studies. Despite using the FATHMM score as a feature in training our model, LYRUS did not exhibit inflated performance (Supplementary Fig. S18). The Spearman’s correlations between FATHMM’s predictions and the experimental results of three PTEN DMS datasets were 0.046, 0.219 and 0.242 (Fig. 4). On the other hand, Spearman’s correlations between FATHMM’s predictions and the experimental results of three TP53 DMS datasets were 0.731, 0.460 and 0.481 (Fig. 5). Undoubtedly, there is a drastic difference in prediction accuracy between PTEN and TP53 DMS datasets against which FATHMM were benchmarked. However, the Spearman’s correlations between LYRUS and six DMS measurements were 0.422, 0.434, 0.467, 0.568, 0.451 and 0.476 (Supplementary Fig. S18). The results exhibited both consistency and accuracy. Some VEPs, such as UNEECON and REVEL, despite achieving notable correlations with the PTEN and TP53 experimental DMS scores, could not generate predictions for SAVs that could not be produced via single nucleotide changes, thereby reducing prediction coverage by 70% (Supplementary Figs S19 and S20). Among six independent DMS performance assessments, the two most widely used VEPs, PolyPhen-2 and SIFT, did not show exceptional performance against the PTEN and TP53 DMS datasets. However, it is interesting that one of the VEPs we assessed, Envision (Gray *et al.*, 2018), was trained with a supervised learning approach using DMS data rather than labeled pathogenic and benign variants from any human mutation databases. Despite this advantage, Envision only had a moderate performance on the PTEN and TP53 DMS datasets, consistent with the results in Livesey *et al.* However, when evaluating methods by the numerical difference between experimental and predicted variant effect scores [mean squared error (MSE)], Envision performed best, immediately followed by PROVEAN (Supplementary Figs S21–S26). Envision’s low MSE was primarily attributed to the overall distribution of prediction scores resembling the distribution of DMS experimental scores (Supplementary Figs S21–S26). LYRUS and other ML VEPs exhibited distributions skewed toward the high effect, indicating better recognition of high effect SAVs. LYRUS was trained on binary classification data (benign or pathogenic); nevertheless, these comparisons have shown that its predictions correlate strongly with effect strength. To a degree, the DMS data replicated this finding, highlighting that even methods trained for classification capture aspects of effect strength.

## 4 Discussion

This study introduces LYRUS, an ML approach with the optimal pipeline selected by TPOT for predicting the pathogenicity of human SAVs. We aimed to develop an algorithm for predicting the clinical pathogenicity of human SAVs, thus, the ClinVar database was used to generate the training dataset. Most methods in the field, such as PolyPhen2 and FATHMM, were designed to predict the effects of SAVs on protein function rather than their clinical significance (Adzhubei *et al.*, 2010; Shihab *et al.*, 2013). Functional effects and clinical significance are not one and the same. However, in order to compare the predictions across a wider range of methods, we purposefully disregarded this subtlety. Databases, such as ClinVar, may involve potential bias from human curators, thus, we added the VariBench dataset as well as six additional DMS datasets to test the performance of the model in an unbiased manner.

Four pairs of features used by LYRUS have correlation coefficients higher than 0.5. The wild-type PSIC and ΔPSIC have a correlation of 0.66, which is expected since the model of sequence family evolution that computes the scores was constructed with the assumption that substitution probabilities are position-dependent (Sunyaev *et al.*, 1999). The wild-type PSIC and variation number have a negative correlation of −0.59, which is intuitively reasonable considering that the lower the variation number, the more conserved a given amino acid is at a particular position, and the higher the PSIC score, the more likely this particular amino acid occurs at this position. SASA and mechanical stiffness have a negative correlation of −0.53, because buried residues with less SASA are more resistant to external pulling forces, thus exhibiting high mechanical stiffness.

The XGBoost classifier was picked by TPOT as the best model for our dataset. The XGBoost classifier minimizes data-overfitting issues (Chen and Guestrin, 2016). A large number of false positives are often a consequence of overfitting, and by using the XGBoost classifier, this issue was minimized in LYRUS, as we observed similar classification rates in both the ClinVar and the VariBench dataset. To prevent the Type I and II data circularity issues mentioned in Grimm *et al.* (2015), we removed the SAVs present in the ClinVar training dataset from the VariBench dataset. We also removed those SAVs whose protein structure was present in the ClinVar dataset from the VariBench dataset to minimize data leakage issues. LYRUS achieved the second highest accuracy, specificity, *F*-measure and MCC using the ClinVar dataset. It also has comparable performance to that of other VEPs using the VariBench dataset, as it has the fourth highest accuracy in the VariBench_selected, and fifth highest accuracy in the VariBench_limited datasets. We wanted to point out that there might be overlaps between the VariBench dataset and the training dataset used by other VEPs. Thus, for the VariBench dataset, other VEPs might have an advantage over LYRUS. M-CAP, MVP and SuSPect exhibited better performances than LYRUS benchmarked against the VariBench_selected and VariBench_limited datasets. However, M-CAP and MVP are meta-predictors, which incorporate the prediction scores from many different VEPs and are designed to outperform other supervised and unsupervised learning methods. Meta-predictors, as evaluated by Grimm *et al.*, may show better performance due to the Type I circularity issue, which occurs when the data from the training set are re-used for assessing predictor performance. These predictors may amplify this issue as the various methods they are built from often use different overlapping training sets. Moreover, they both may suffer from overfitting, as illustrated in their inconsistent predictive performances when benchmarked against six DMS datasets. Furthermore, SuSPect may also suffer from overfitting issues as it was trained using 77 predictive features. If too many features were used when training a method without enough training data, the learned hypothesis might fit the training dataset well but fail to generalize to new examples (Ying, 2019). This can be seen in SuSPect’s strong correlation between its predictions and PTEN DMS experimental values but weak correlations between its predictions and TP53 DMS experimental values. Additionally, FATHMM may suffer from label leakage issues shown in its divergent prediction accuracy benchmarked against PTEN DMS datasets compared to against TP53 datasets. This finding was consistent with the results presented in Grimm *et al.* (2015).

The most predictive features in LYRUS are sequence-based features. Studies have shown the importance of using amino acid conservation for pathogenicity prediction, which explains the high impact of sequence-based features in LYRUS (Adzhubei *et al.*, 2010; Bromberg *et al.*, 2008; Choi *et al.*, 2012). The high impact score of the variation number also suggests the effectiveness of this novel feature for categorizing pathogenic and non-pathogenic SAVs. Although structural and dynamics-based features have lower weights in LYRUS, these features are still valuable to include. Existing studies have shown that combining information gained from multiple sequence alignment and 3D protein structures increases prediction performance (Bromberg *et al.*, 2008; Saunders and Baker, 2002). Among the structural and dynamic features, changes in folding free energies and the location of binding sites have the highest weights in LYRUS. In fact, several computational approaches have been developed to predict ΔΔ*G*_fold_ in order to link it to the pathogenicity of mutations, which suggests the importance of incorporating ΔΔ*G*_fold_ into LYRUS (Peng and Alexov, 2016; Petukh *et al.*, 2015). Catalytic residues, which comprise drug binding sites, are often conserved during evolution, and mutations of these residues can be detrimental (Porter *et al.*, 2004). This suggests the importance of incorporating information regarding the location of binding sites into pathogenicity predictors.

Although studies have demonstrated the utility of considering the equilibrium dynamics of proteins as a mean of improving the predictive ability of pathogenicity predictors, our study reveals that dynamics-based features did not significantly contribute to the predictive power of LYRUS. One reason that dynamics-based features have a low impact score in LYRUS might be the limitations imposed by the models we used to calculate them. For example, the main disadvantage of the anisotropic network model is its inability to account for anharmonic motions or multimeric transitions driven by a protein’s slowest collective modes (Doruker *et al.*, 2000). The use of more sophisticated dynamics models may better capture the protein dynamics and further improve the prediction accuracy. The inclusion of other dynamic models is of future interest.

Another area for future improvement is the incorporation of structural changes caused by mutations into the model. LYRUS predicts the pathogenicity of SAVs based on the original protein structure instead of the mutated one. It has been proven that mutations promoting protein misfolding contribute to a variety of human diseases. Incorporating information related to structural changes, such as protein root mean square deviations, which reflect structural changes, may facilitate pathogenicity prediction (Doss and Zayed, 2017; Mishra *et al.*, 2017; Studer *et al.*, 2013). Other thermodynamic information, such as changes in binding free energies, may also enhance the accuracy of the model. The prediction method may additionally be extended to other types of DNA mutations, such as insertions and deletions, which may result in frameshifts.

In this study, a small part of the PTEN structure was simulated. However, because our method relies heavily on the PDB structure of the protein, we would not recommend applying LYRUS to a protein whose experimental PDB structure is unavailable. Thus, our method cannot be applied to proteins, such as BRCA1, which is a limitation of our approach. The inability to predict some variants are due to the lack of the experimental structures. With advances in protein folding algorithms, such as AlphaFold, it may become possible to predict the pathogenicity of SAVs using predicted structures (Senior *et al.*, 2020). Future work is needed to generate a pipeline, which can be applied to simulated structures. LYRUS is built upon existing software (Supplementary Table S2), and the most computationally expensive part of the method is the calculation of ΔΔ*G*_fold_ using FoldX.

Many DMS experiments included SAVs that are not accessible via single nucleotide changes. Some VEPs, such as UNEECON, REVEL, M-CAP, MVP, MutationAssessor and PrimateAI, do not produce predictions for these variants. Some VEPs incorporate features derived from experimentally determined protein structures into their predictions. Rhapsody, for instance, requires a PDB structure to make its predictions and uses features representing the 3D environment of the mutation. In the event that a PDB structural file is lacking or the structure does not span the entire protein, the software will either fail to generate results or generate incomplete results (i.e. no prediction will be yielded for certain SAVs). As inferred by Grimm *et al.*, FATHMM’s apparently exceptional performance observed when benchmarked using Grimm *et al.*’s *VariBench* was likely due to Type 2 circularity-associated inflation (Grimm *et al.*, 2015). Despite incorporating the FATHMM score as a feature in training our model, LYRUS did not suffer from the same issue. One noticeable challenge for DMS is the development of an assay to measure the functional impact (Starita *et al.*, 2017). Evaluating proteins with multiple functions requires multiple assays. Even for the same assay, specific experimental conditions might influence measurements (Melnikov *et al.*, 2014). In addition, variants that could affect molecular function as assayed by DMS are sometimes clinically classified as benign. Considering all of the above and the fact that LYRUS was benchmarked against pathogenic human mutation datasets, LYRUS still consistently produced excellent performance for a total of six PTEN and TP53 DMS datasets.

## 5 Conclusion

This study presents an ML pipeline (LYRUS) for predicting human SAV pathogenicity that incorporates variation number along with 14 other features. LYRUS attained an accuracy of 0.859 using an autoML-selected (TPOT) XGBoost classifier. The XGBoost model suggests that sequence-based features have larger weights than structural and dynamic features in SAV pathogenicity prediction. The variation number, a unique feature, we employed, is negatively correlated with clinical pathogenicity, and has the fourth highest weight among all of the features studied here. Performance analysis using both the ClinVar and VariBench datasets showed that LYRUS performed comparably to the best of 13 other VEPs that were benchmarked in this work. Performance analysis using PTEN and TP53 DMS datasets showed that LYRUS consistently exhibited strong predictive power. The scripts for LYRUS are available at https://github.com/jiaying2508/LYRUS.

## Supplementary Material

vbab045_Supplementary_Data
