# Supplementary material for: LYRUS: a machine learning model for predicting the pathogenicity of missense variants
Source: Bioinform Adv. 2021 Dec 25;2(1):vbab045. doi: 10.1093/bioadv/vbab045 (PMC8754197; doi:10.1093/bioadv/vbab045)
Supplement: vbab045_Supplementary_Data [file vbab045_supplementary_data.pdf]

Supplemental Information for "LYRUS: A Machine Learning Model for Predicting the Pathogenicity of Missense Variants"

Jiaying Lai[1,2†], Jordan Yang[3†], Ece D. Gamsiz Uzun[2,4,5], Brenda M. Rubenstein[2,3*], Indra Neil Sarkar[1,6*],

1 Center for Biomedical Informatics, Brown University, Providence, Rhode Island, United States of America
2 Center for Computational Molecular Biology, Brown University, Providence, Rhode Island, United States of America
3 Department of Chemistry, Brown University, Providence, Rhode Island, United States of America
4 Department of Pathology and Laboratory Medicine, Brown University Alpert Medical School, Providence, Rhode Island, United States of America
5 Department of Pathology, Rhode Island Hospital, Providence, Rhode Island, USA
6 Rhode Island Quality Institute, Providence, Rhode Island, United States of America

* To whom correspondence should be addressed.
† These authors contributed equally to this work.

Table S1. SAV counts for the ClinVar and VariBench datasets.

Datasets	Pathogenic Variants (P)	Benign Variants (B)	Total	Ratio (P:B)
ClinVar	11920	10719	22639	1.11
VariBench_selected	3466	4757	8223	0.73
VariBench_limited	2886	2009	4895	1.44

Source
ClinVar: https://ftp.ncbi.nlm.nih.gov/pub/clinvar
VariBench_selected: http://structure.bmc.lu.se/VariBench/GrimmDatasets.php
VariBench_limited: http://structure.bmc.lu.se/VariBench/GrimmDatasets.php

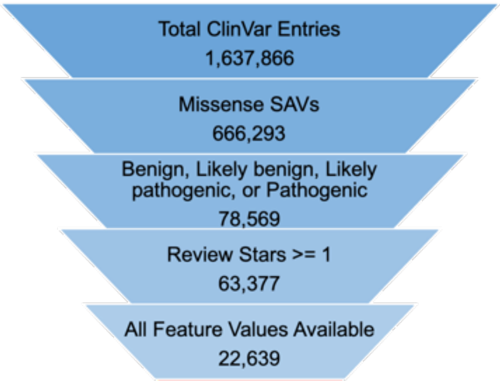

Figure S1. Number of SAVs from ClinVar. Among all of the SAVs available in ClinVar, roughly 1.4% of SAVs meet the selection criteria. Among the selected SAVs, 10719 SAVs (47%) are benign and 11920 SAVs (53%) are pathogenic.

Table S2. Links to the software used to compute each feature.

Feature Name	Link
Variation Number	https://github.com/jiaying2508/variation_number
ΔE Epistatic Score	https://github.com/debbiemarkslab/EVmutation
FIS	http://fathmm.biocompute.org.uk
ΔPSIC	http://genetics.bwh.harvard.edu/pph2/
Wild-type PSIC	http://genetics.bwh.harvard.edu/pph2/
$\Delta\Delta G_{fold}$	http://foldxsuite.crg.eu
SASA	https://freesasa.github.io
Mutant SSF	https://pbwww.che.sbg.ac.at/?page_id=416
Active Site Value	https://github.com/rdk/p2rank
Mutant Reference Energy	http://www.pyrosetta.org
ΔReference Energy	http://www.pyrosetta.org
MSD	http://prody.csb.pitt.edu
Mechanical Stiffness	http://prody.csb.pitt.edu
Effectiveness	http://prody.csb.pitt.edu
Sensitivity	http://prody.csb.pitt.edu

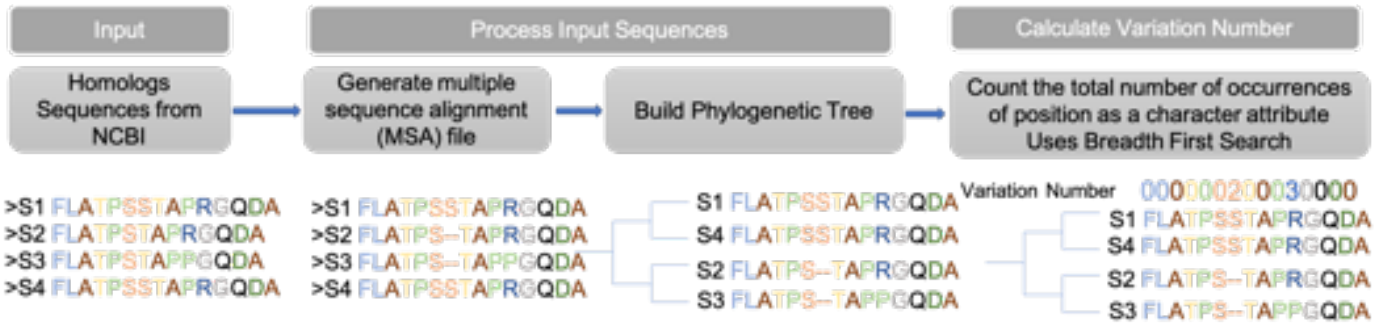

Figure S2. Pipeline for Calculating Variation Number. Variation number counts the number of occurrences of a position as a character attribute in all tree clades, where a character attribute may be defined as a state that exists in some elements of a clade, but not in the alternate clade under the same parent node. For a given amino acid sequence, the orthologous sequences are obtained using the NCBI Orthologs Database. Multiple sequence alignment files are built using Clustal Omega. Phylogenetic trees are generated using PAUP software with the maximum parsimony method. Variation number, calculated using breadth first search, is the number of occurrences of a position as a character attribute in a given tree. For each amino acid sequence, variation numbers at all of the human positions are normalized using min-max normalization. A smaller variation number suggests more conservation. The software is available at https://github.com/jiaying2508/variation_number.

Table S3. Summary of the other VEPs assessed in this study and a description of predictive features Incorporated in their methods.

VEP	Category	Features	Data source, method source, or online website
PolyPhen-2	Supervised	Sequence conservation, sequence features, residue-level structural features	http://genetics.bwh.harvard.edu/pph2/
PROVEAN	Unsupervised	Sequence conservation	http://provean.jcvi.org/seq_submit.php
SIFT	Empirical	Sequence conservation	http://provean.jcvi.org/seq_submit.php
Rhapsody	Supervised	Sequence features, structural features, Elastic network model related features	http://rhapsody.csb.pitt.edu/
EVmutation	Unsupervised	sequence co-variation	http://rhapsody.csb.pitt.edu/ /evmutation/downloads.html
MutationAssessor	Unsupervised	Sequence conservation, conservation between subfamilies	http://mutationassessor.org/r3/
SuSPect	Supervised	Network centrality, uniprot annotations, sequence conservation, SASA	http://www.sbg.bio.ic.ac.uk/suspect/
FATHMM	Supervised	HMM alignments, per-domain mutation consequences	http://fathmm.biocompute.org.uk/inherited.html
MVP	Metapredictor	Eigen, VEST3, MutationTaster, PolyPhen-2, SIFT,PROVEAN,FATHMM, MutationAssessor,LRT	https://github.com/ShenLab/missense
PrimateAI	Supervised	Sequence features,structural features	https://github.com/Illumina/PrimateAI
UNEECON	Supervised	sequence conservation scores, protein structural features, functional genomic features	https://github.com/yifei-lab/UNEECON
M-CAP	Metapredictor	SIFT,PolyPhen-2,CADD,MetaLR,MutationTaster, MutationAssessor,FATHMM,LRT, evolutionary conservation metrics, substitution matrices	http://bejerano.stanford.edu/mcap/
REVEL	MetaPredictor	MutPred, PROVEAN, SIFT, PolyPhen-2,LRT, MutationTaster,MutationAssessor,FATHMM VEST3,GERP++,SiPhy,PhvloP	https://sites.google.com/site/revelgenomics/
Envision	Supervised	DMS measurements	https://envision.gs.washington.edu /shiny/envision_new/

Table S4. VEP cutoffs between benign and pathogenic variants. 'Used by' means that the cutoff was used in the source. 'Author recommendation' means that the cutoff was recommended by the author. The cutoffs for MVP and UNEECON were calculated by using the threshold that minimizes the difference between the true positive rate and 1 - false the positive rate in the VariBench_selected dataset.

VEP	Cutoff	Reason
PolyPhen-2	0.453	Used by Polyphen2
PROVEAN	-2.5	Used by PROVEAN
SIFT	0.05	Used by PROVEAN
Rhapsody	0.5	Used by Rhapsody
EVmutation	-4.75	Used by Rhapsody
MutationAssessor	1.94	Author recommendation
SuSPect	50	Author recommendation
FATHMM	-1.5	Author recommendation
MVP	0.83	Calculated
PrimateAI	0.6	Author recommendation
M-CAP	0.95	Author recommendation
REVEL	0.5	Author recommendation
UNEECON	0.16	Calculated

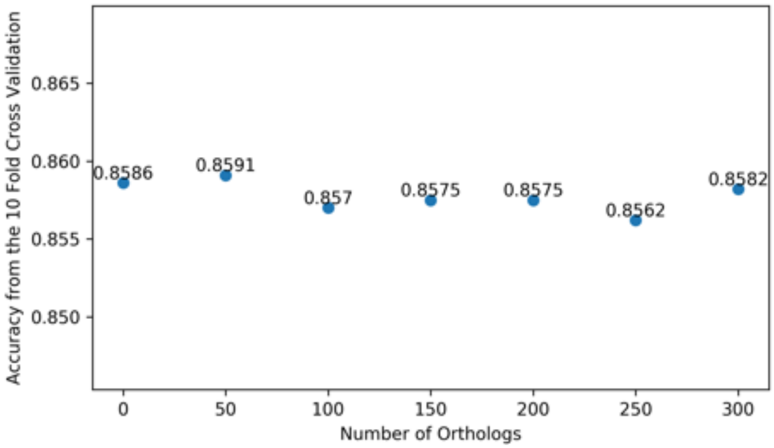

Figure S3. Model Accuracy Using Different Numbers of Orthologs. Different models were trained using SAVs with at least 0, 50, 100, 150, 200, 250, or 300 orthologous sequences. Each model was assessed using the ClinVar dataset and the accuracy was estimated using 10-fold cross-validation. The accuracy of each model is similar.

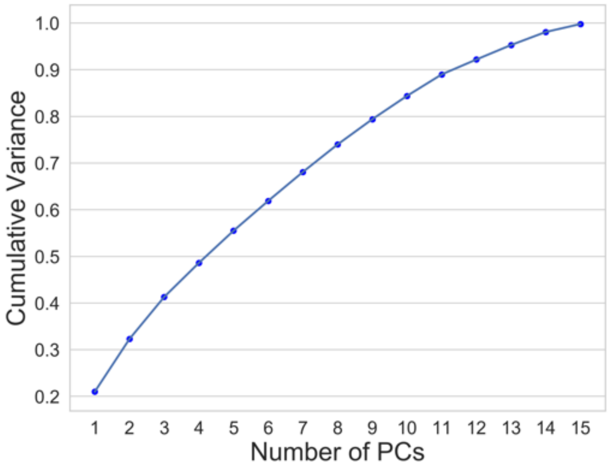

Figure S4. PCA. Plot of the cumulative variance vs. the number of principal components from a PCA analysis of our 15 features. This cumulative variance plot illustrates that 13 components are needed to describe 90% of the variance in the data.

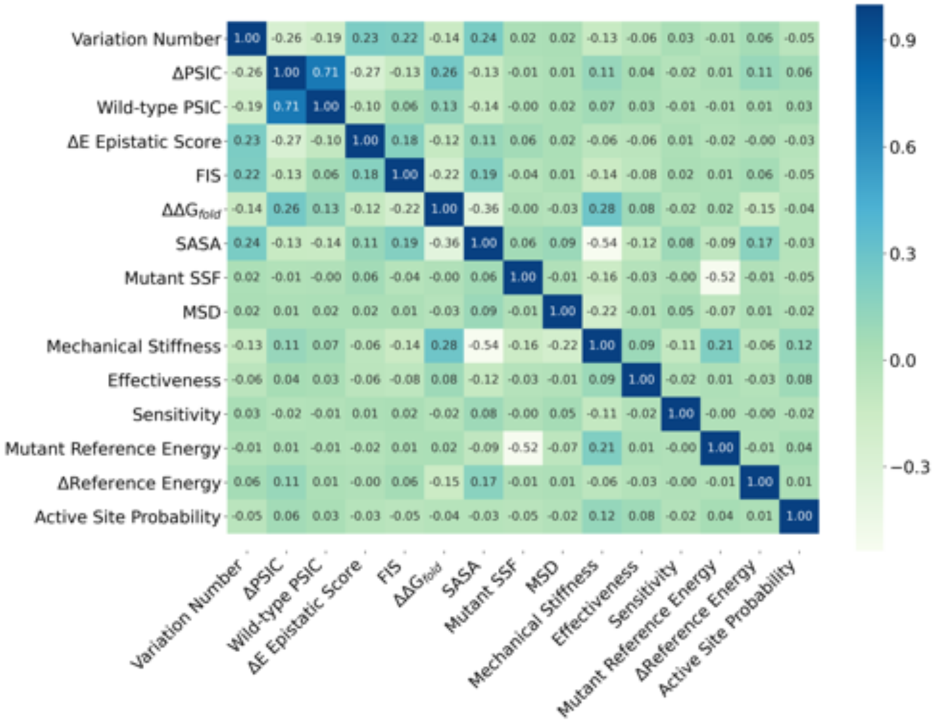

(a) Pearson Correlation Coefficient Heatmap

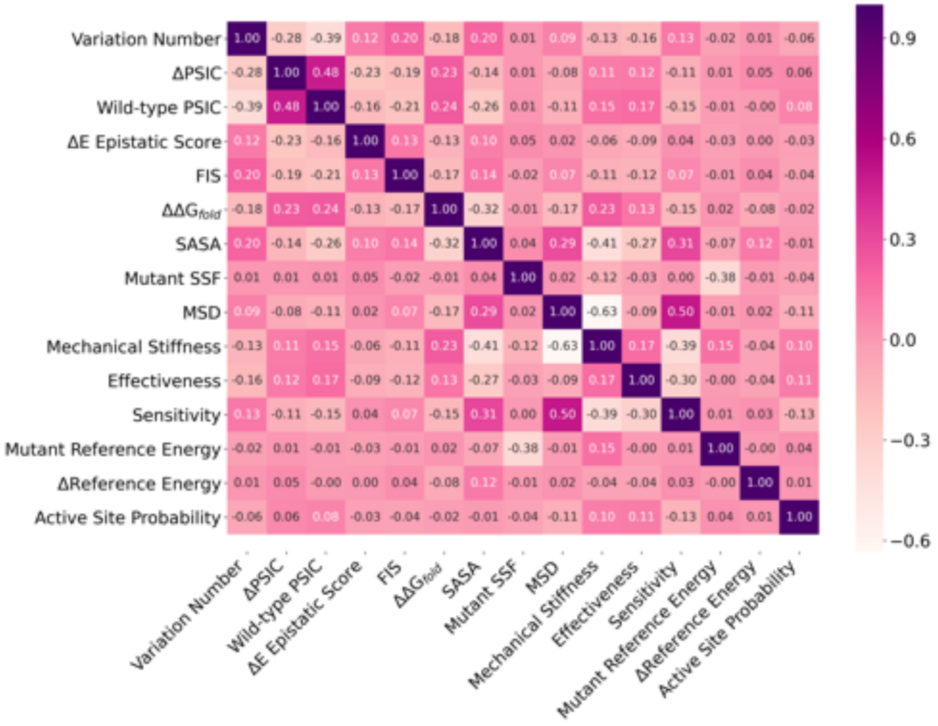

(b) Kendall Rank Correlation Coefficient Heatmap

Figure S5. Feature Correlation Heatmap. (a) Pearson's r calculated between all possible pairwise combinations of the 15 features. Out of the 105 possible pairs of feature combinations, only three pairs had a correlation coefficient (absolute value) greater than 0.4, suggesting that almost all (97%) possible pairwise feature combinations did not exhibit a significant linear relationship. (b) Kendall's Tau calculated between all possible pairwise combinations of the 15 features. Out of the 105 possible pairs of feature combinations, only seven pairs had a coefficient (absolute value) greater than 0.35, suggesting that 93% of all possible pairwise feature combinations did not exhibit a strong monotonic relationship.

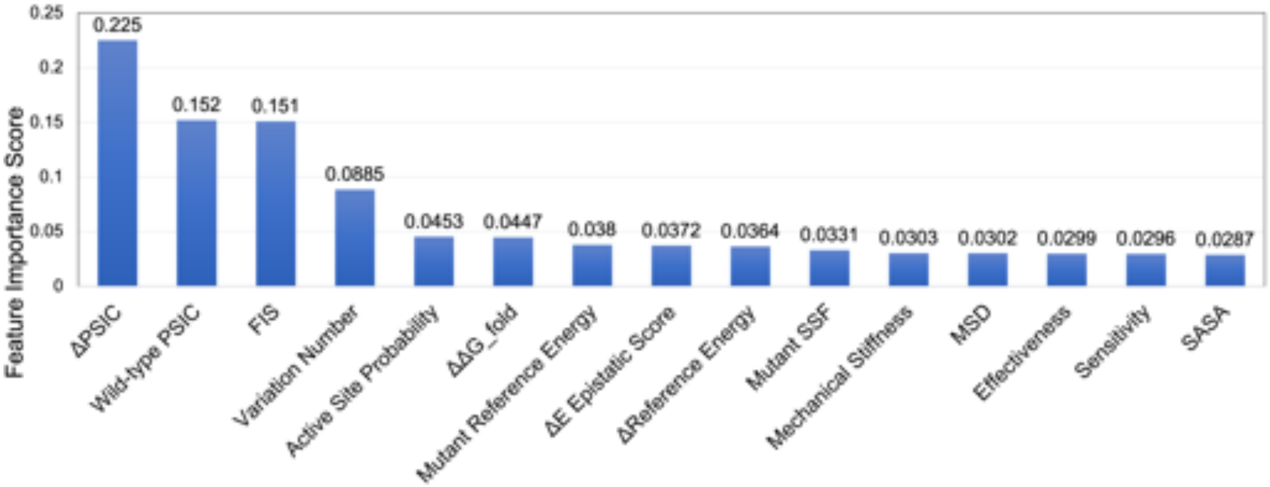

Figure S6. Feature Importance Scores from LYRUS. Feature importance scores were calculated using the built-in function "feature_importances_" from the xgboost package in Python. Given the stochastic nature of the xgboost model, feature importance scores vary for each run, and the average of 1000 repeats were taken as the feature importance scores shown here. The sequence-based features had higher weights than structural and dynamics-based features. ΔPSIC had the highest importance score, followed by the wild-type PSIC, FIS, and variation number. The remaining 11 features had similar importance scores.

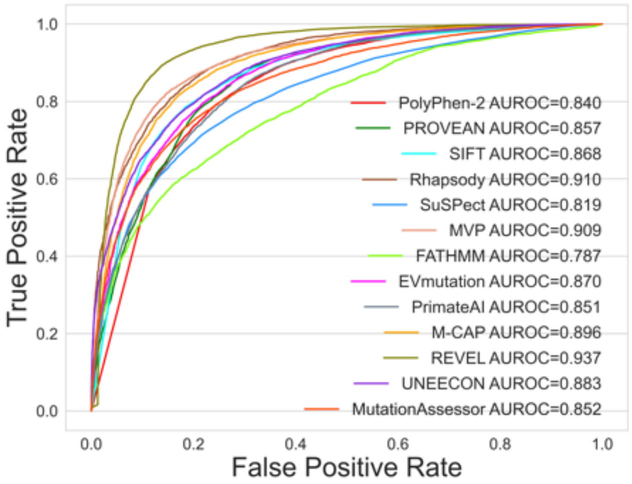

(a) Area under the ROC Curve (AUC)

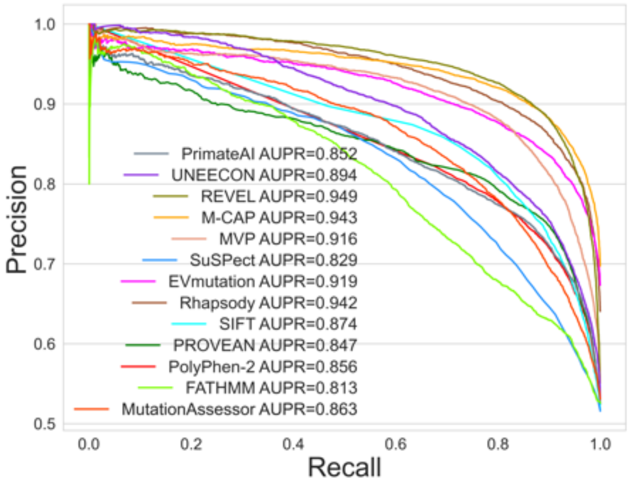

(b) Area under the PR Curve (AUPR)

Figure S7. Comparison of ROC and PR Curves of the 13 other VEPs from the ClinVar dataset. (a) ROC curves. (b) PR curves.

Table S5. Performance analysis of LYRUS and 13 other VEPs tested on the ClinVar dataset. MCC = Matthews Correlation Coefficient.

Model Name	Accuracy	Sensitivity	Specificity	F-measure	MCC
LYRUS	0.859	0.860	0.857	0.866	0.716
PolyPhen-2	0.743	0.915	0.571	0.781	0.518
PROVEAN	0.784	0.890	0.679	0.805	0.582
SIFT	0.770	0.903	0.638	0.797	0.561
Rhapsody	0.820	0.806	0.84	0.843	0.636
EVMutation	0.785	0.806	0.748	0.829	0.543
MutationAssessor	0.744	0.859	0.630	0.770	0.502
SuSPect	0.736	0.592	0.876	0.688	0.489
FATHMM	0.724	0.755	0.694	0.729	0.449
MVP	0.691	0.985	0.363	0.77	0.453
PrimateAI	0.767	0.795	0.74	0.774	0.536
MCAP	0.784	0.986	0.372	0.859	0.497
REVEL	0.866	0.937	0.795	0.875	0.739
UNEECON	0.781	0.831	0.729	0.793	0.564

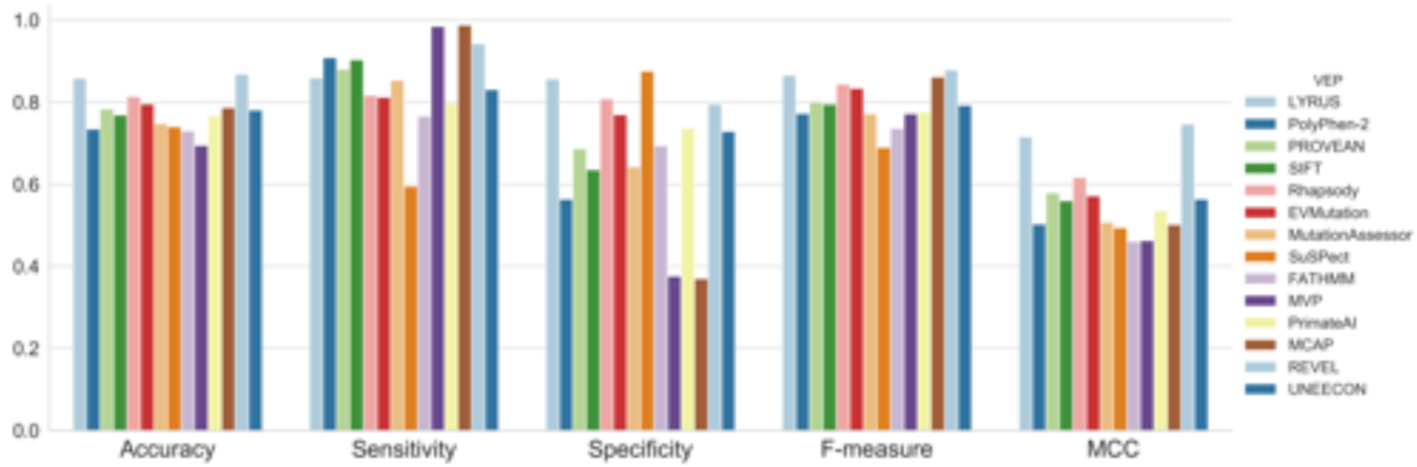

Figure S8. LYRUS Statistics Compared to Those of Other VEPs When Tested on the ClinVar Dataset. The accuracy, sensitivity, specificity, F-measure, and MCC for each of the prediction methods were calculated. LYRUS achieved the second highest accuracy, specificity, F-measure, and MCC.

Table S6. Performance analysis of LYRUS and 12 other VEPs using the VariBench_selected dataset.

Model Name	TP	TN	FP	FN	Accuracy	Sensitivity	Specificity	F-measure	MCC
LYRUS	2581	4023	734	885	0.803	0.745	0.846	0.761	0.594
PolyPhen-2	2432	2310	2446	1034	0.577	0.702	0.486	0.583	0.188
PROVEAN	1828	2528	1615	1421	0.589	0.563	0.61	0.546	0.172
SIFT	2214	2125	1983	958	0.596	0.698	0.517	0.601	0.216
EVmutation	1180	614	432	755	0.602	0.61	0.587	0.665	0.188
MutationAssessor	2219	2441	1624	1163	0.626	0.656	0.6	0.614	0.256
SuSPect	2714	3630	589	550	0.848	0.831	0.86	0.827	0.691
FATHMM	3184	3711	606	251	0.889	0.927	0.86	0.881	0.782
MVP	3028	3475	516	421	0.874	0.878	0.871	0.866	0.747
PrimateAI	1520	3337	1241	1747	0.619	0.465	0.729	0.504	0.2
MCAP	3361	1503	1364	61	0.773	0.982	0.524	0.825	0.584
REVEL	2523	4043	707	943	0.799	0.728	0.851	0.754	0.586
UNEECON	2227	3005	1641	1239	0.645	0.643	0.647	0.607	0.287

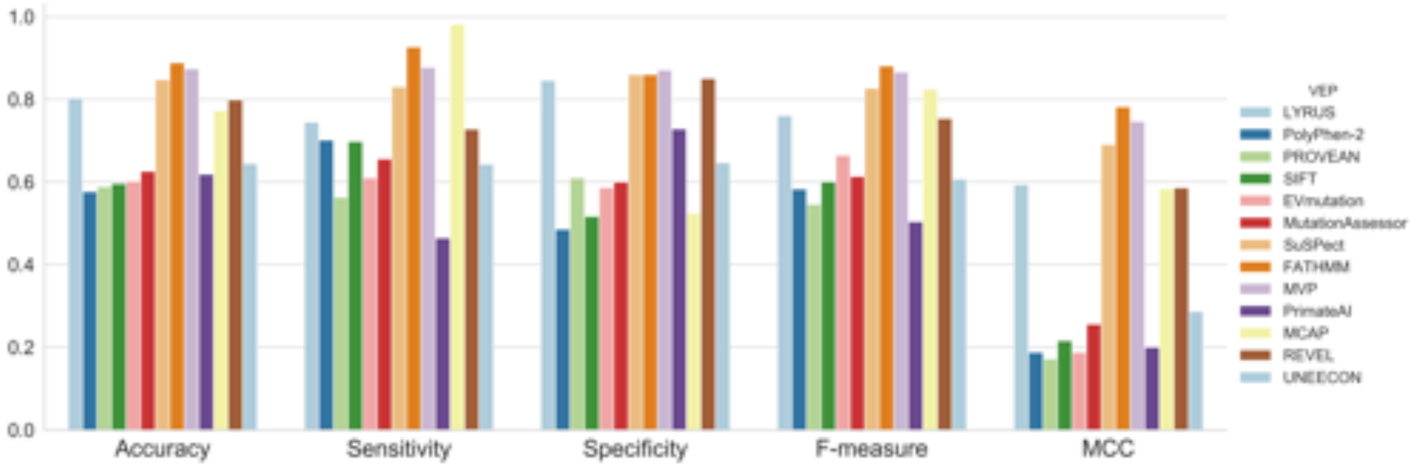

Figure S9. LYRUS Statistics Compared to Those of Other VEPs When Test on the VariBench_selected Dataset. The accuracy, sensitivity, specificity, F-measure, and MCC for each of the prediction methods were shown.

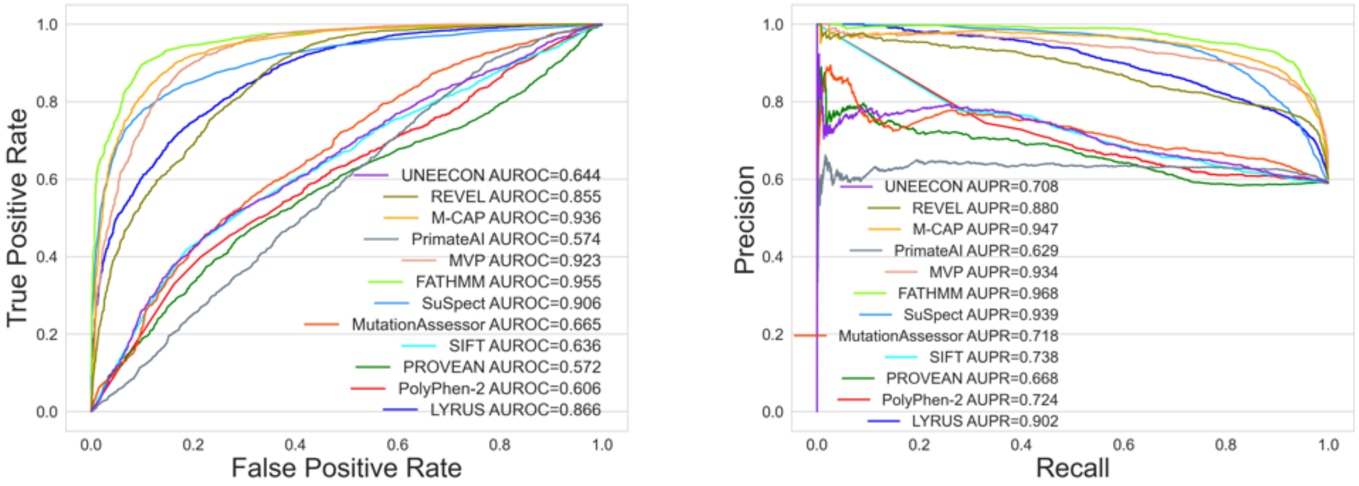

(a) Area under the ROC Curve (AUC) **(b)** Area under the PR Curve (AUPR)

Figure S10. Comparison of ROC and PR Curves of 12 VEPs from the VariBench_limited Dataset. (a) ROC curves. (b) PR curves.

Table S7. Comparison to Other VEPs using VariBench_limited.

Model Name	TP	TN	FP	FN	Accuracy	Sensitivity	Specificity	F-measure	MCC
LYRUS	2164	1577	432	722	0.764	0.75	0.785	0.789	0.527
PolyPhen-2	2013	835	1174	873	0.582	0.698	0.416	0.663	0.117
PROVEAN	1683	1080	929	1203	0.564	0.583	0.538	0.612	0.119
SIFT	2035	933	1076	851	0.606	0.705	0.464	0.679	0.173
MutationAssessor	1963	1071	938	923	0.62	0.68	0.533	0.678	0.214
SuSPect	2426	1653	356	460	0.833	0.841	0.823	0.856	0.659
FATHMM	2702	1683	326	184	0.896	0.936	0.838	0.914	0.784
MVP	2506	1673	336	380	0.854	0.868	0.833	0.875	0.699
PrimateAI	1261	1292	717	1625	0.522	0.437	0.643	0.519	0.08
MCAP	2841	1029	980	45	0.791	0.984	0.512	0.847	0.59
REVEL	2079	1571	438	807	0.746	0.72	0.782	0.77	0.494
UNEECON	1891	1078	931	995	0.607	0.655	0.537	0.663	0.191

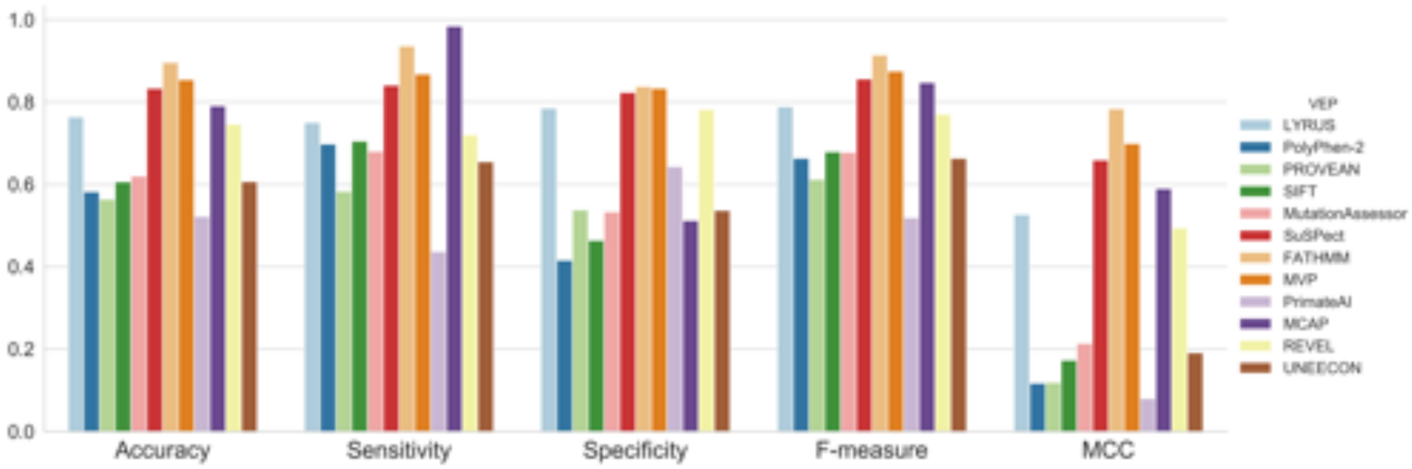

Figure S11. Statistics Compared to Other Software using VariBench_limited. The accuracy, sensitivity, specificity, F-measure, and MCC for each of the prediction methods are shown.

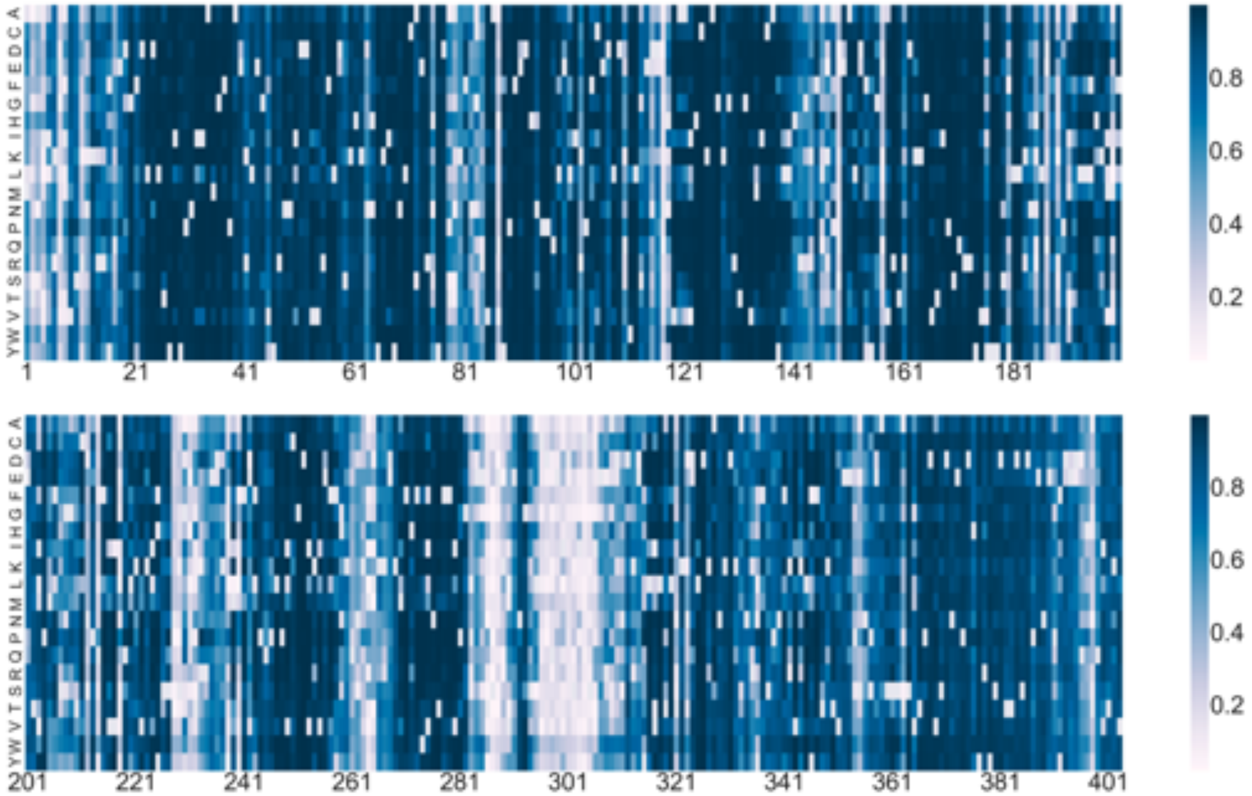

Figure S12. PTEN Prediction Heatmap. The x-axis represents PTEN amino acid positions and the y-axis represents different amino acid substitutions. The color coding of each heatmap cell represents the predicted probability of the SAV being pathogenic. Wild-type amino acids were assigned a probability of 0. LYRUS predicts most PTEN SAVs to be pathogenic.

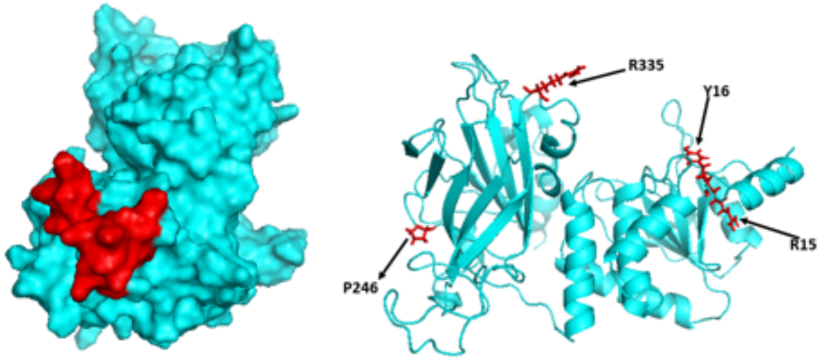

(a) **(b)**

Figure S13. Cartoon and Surface Representations of the PTEN Protein. (a) The red surface corresponds to PTEN positions 286 to 305. (b) Visualization of the positions of the four false negative variants.

Table S8. PTEN Case Study. A total of 110 SAV classifications are available from ClinVar. True positive (TP), true negative (TN), false positive (FP), false negative (FN), no prediction (NP), accuracy, sensitivity, specificity, F-measure, and MCC are listed.

Model Name	TP	TN	FP	FN	Accuracy	Sensitivity	Specificity	F-measure	MCC
LYRUS	104	2	0	4	0.964	0.963	1.0	0.981	0.567
PolyPhen-2	98	2	0	10	0.909	0.907	1.0	0.951	0.389
PROVEAN	103	2	0	5	0.955	0.954	1.0	0.976	0.522
SIFT	102	2	0	6	0.945	0.944	1.0	0.971	0.486
Rhapsody	88	2	0	20	0.818	0.815	1.0	0.898	0.272
EVMutation	105	0	2	3	0.955	0.972	0.0	0.977	-0.023
MutationAssessor	102	2	0	6	0.945	0.944	1.0	0.971	0.486
SuSPect	108	1	1	0	0.991	1.0	0.5	0.995	0.704
FATHMM	108	0	2	0	0.982	1.0	0.0	0.991	N/A
MVP	108	0	2	0	0.982	1.0	0.0	0.991	N/A
PrimateAI	108	0	2	0	0.982	1.0	0.0	0.991	N/A
M-CAP	108	0	2	0	0.982	1.0	0.0	0.991	N/A
REVEL	108	1	1	0	0.991	1.0	0.5	0.995	0.704
UNEECON	106	2	0	2	0.982	0.981	1.0	0.991	0.701

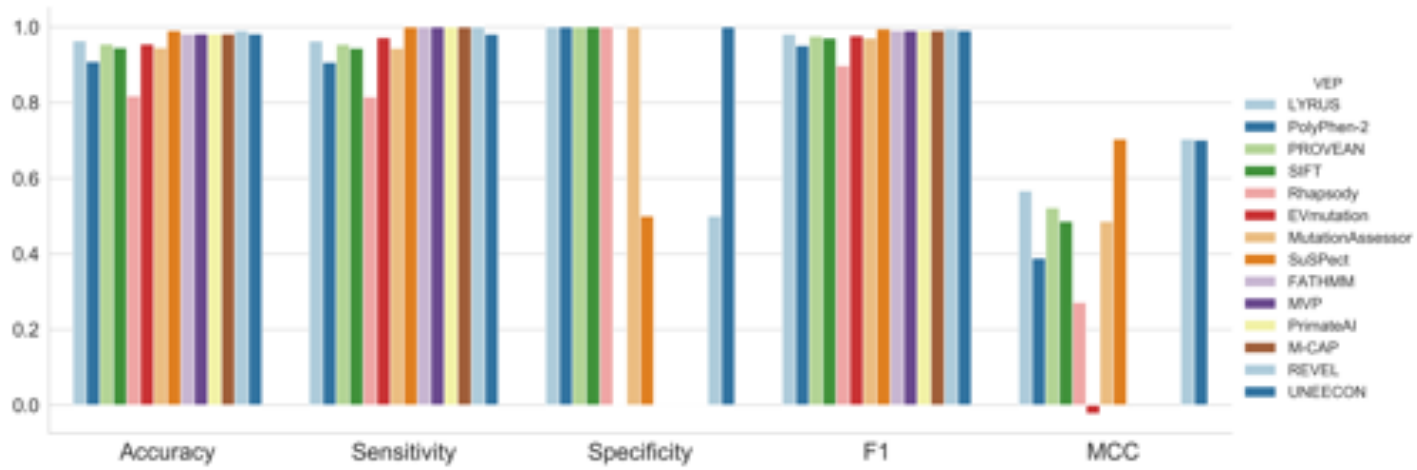

Figure S14. LYRUS Statistics Compared to Those of Other Software When Tested on PTEN. The accuracy, sensitivity, specificity, F-measure, and MCC are shown.

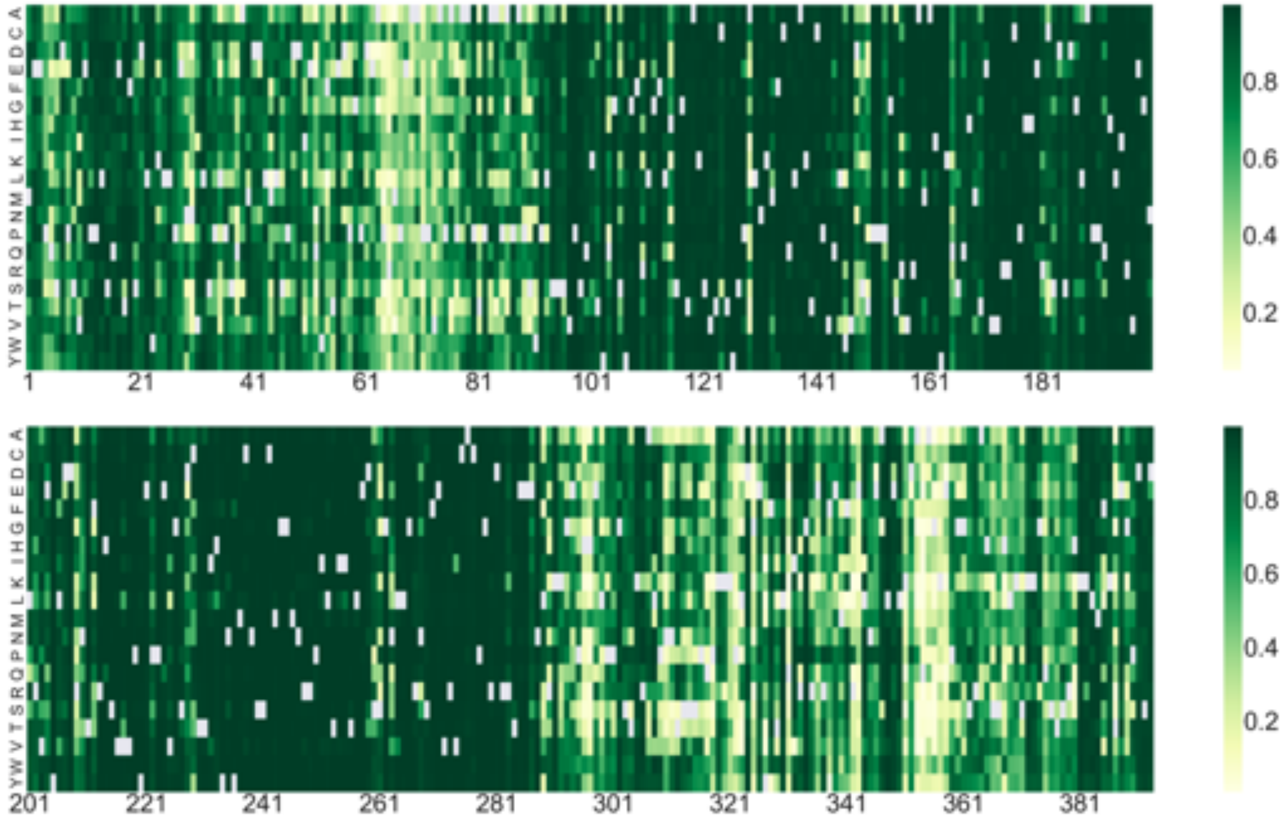

Figure S15. TP53 Prediction Heatmap. The x-axis represents TP53 amino acid positions and the y-axis represents different amino acid substitutions. The color coding of each heatmap cell represents the predicted probability of the SAV being pathogenic. Wild-type amino acids were assigned a probability of 0.

Table S9. TP53 Case Study. A total of 142 SAV classifications are available from ClinVar. True positive (TP), true negative (TN), false positive (FP), false negative (FN), accuracy, sensitivity, specificity, F-measure, and MCC are listed.

Model Name	TP	TN	FP	FN	Accuracy	Sensitivity	Specificity	F-measure	MCC
LYRUS	128	7	6	1	0.951	0.992	0.539	0.973	0.664
PolyPhen-2	125	13	0	4	0.972	0.969	1.0	0.984	0.861
PROVEAN	122	12	1	7	0.944	0.946	0.923	0.968	0.736
SIFT	127	12	1	2	0.979	0.984	0.923	0.988	0.878
Rhapsody	129	6	7	0	0.951	1.0	0.462	0.974	0.662
EVmutation	107	9	4	22	0.817	0.829	0.692	0.892	0.364
MutationAssessor	127	12	1	2	0.979	0.984	0.923	0.988	0.878
SuSPect	110	1	12	19	0.782	0.853	0.077	0.876	-0.058
FATHMM	129	0	13	0	0.908	1.0	0.0	0.952	N/A
MVP	129	0	13	0	0.908	1.0	0.0	0.952	N/A
PrimateAI	111	11	2	18	0.859	0.86	0.846	0.917	0.505
M-CAP	129	0	13	0	0.908	1.0	0.0	0.952	N/A
REVEL	129	6	7	0	0.951	1.0	0.462	0.974	0.662
UNEECON	95	13	0	34	0.761	0.736	1.0	0.848	0.451

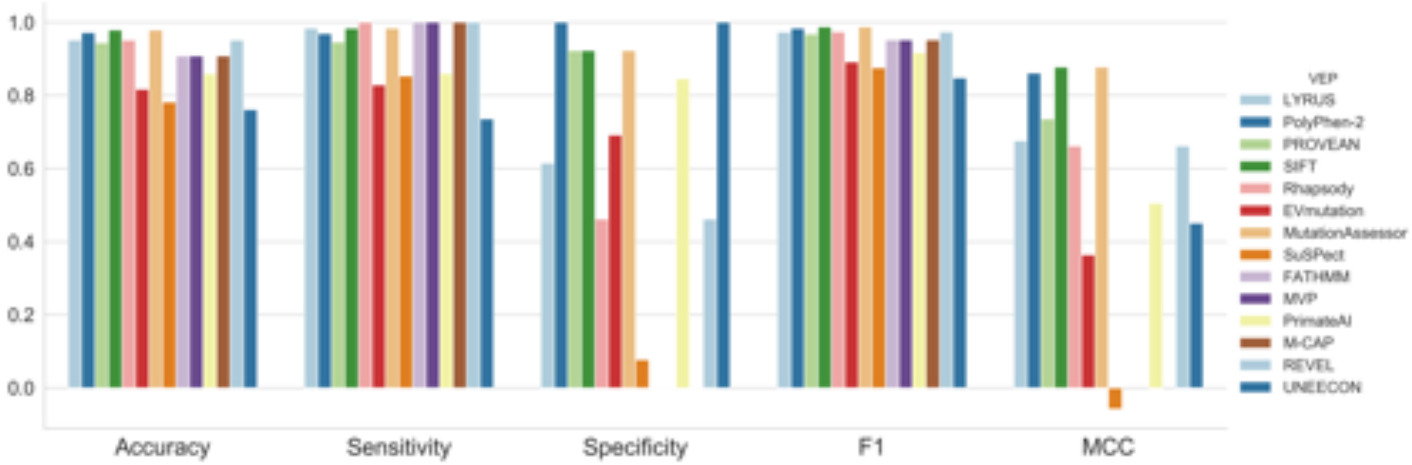

Figure S16. TP53 Statistics Compared to Other Software. The accuracy, sensitivity, specificity, F-measure and MCC are shown.

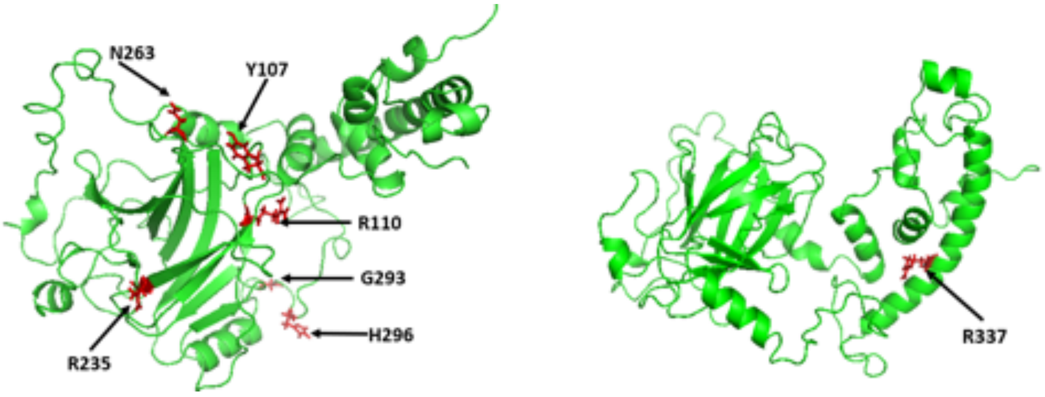

(a) (b)

Figure S17. Carton Representation of the TP53 Protein. (a) Visualization of the positions of the six false positive variants. (b) Visualization of the positions of the one false negative variant.

Table S10. Summary of the DMS datasets used in this study for calculating correlation with the VEP predictions

Dataset	Functional Assay	Mutagenesis Method	Total SAVs	Reference
pten(a)	Site-directed mutagenesis	Fluorescence of a GFP fusion protein	4112	Matreyek *et al.* 2018
pten(b)	Site-directed mutagenesis	Disruption of an artificial genetic circuit in yeast	7264	Mighell *et al.* 2018
pten(highqual_b)	Site-directed mutagenesis	Disruption of an artificial genetic circuit in yeast	6564	Mighell *et al.* 2018
p53(wt_nutlin)	Site-directed mutagenesis	Competitive growth assay in the presence of P53 agonists	7467	Giacomelli *et al.* 2018
p53(null_nutlin)	Site-directed mutagenesis	Competitive growth assay in the presence of P53 agonists	7467	Giacomelli *et al.* 2018
p53(null_etoposide)	Site-directed mutagenesis	Competitive growth assay in the presence of P53 agonists	7467	Giacomelli *et al.* 2018

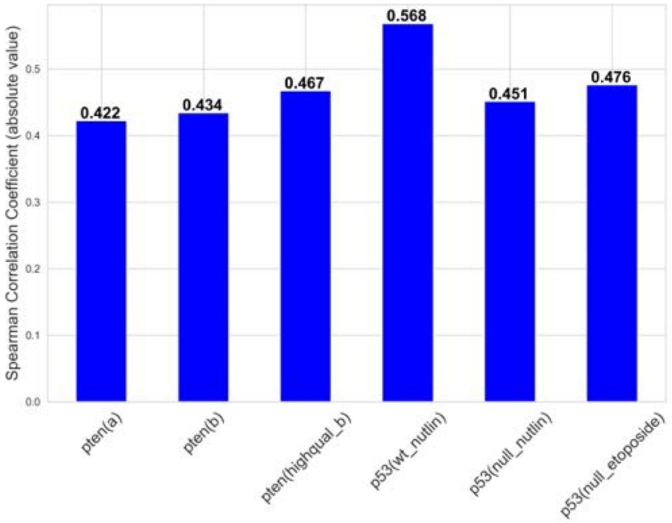

Figure S18. Correlation between LYRUS and DMS Measurements. Spearman's correlation calculated (absolute value) between LYRUS and six DMS datasets.

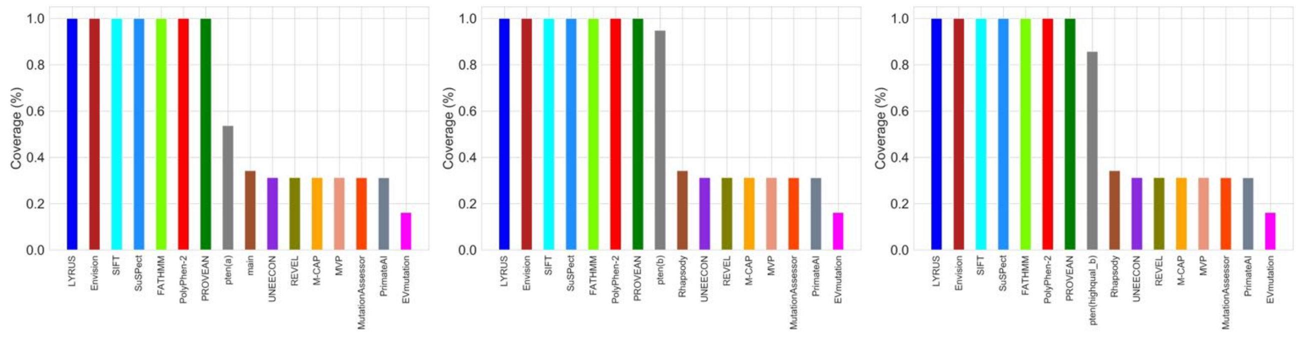

(a) **(b)** **(c)**

Figure S19. Coverage of Possible Mutations by Three PTEN DMS Data sets and 15 VEPs. The percentages of all PTEN amino acid substitutions covered by three PTEN DMS Datasets are colored in gray; the percentages of all PTEN amino acid substitutions covered by each VEP is colored in corresponding colors. (a) coverage of pten(a) and 15 VEPs (b) coverage of pten(b) and 15 VEPs (c) coverage of pten(highqual_b) and 15 VEPs

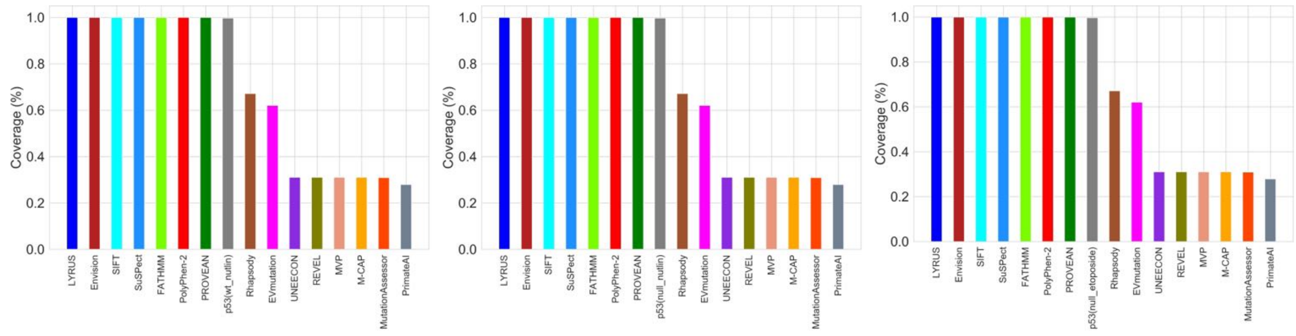

(a) **(b)** **(c)**

Figure S20. Coverage of Possible Mutations by Three TP53 DMS Data sets and 15 VEPs. The percentages of all TP53 amino acid substitutions covered by three TP53 DMS Datasets are colored in gray; the percentages of all TP53 amino acid substitutions covered by each VEP is colored in corresponding colors. (a) coverage of p53(wt_nutlin) and 15 VEPs (b) coverage of p53(null_nutlin) and 15 VEPs (c) coverage of p53(null_etoposide) and 15 VEPs

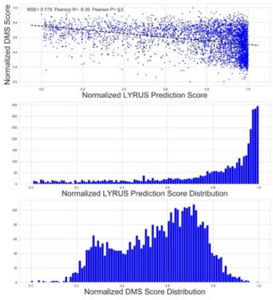

(a)

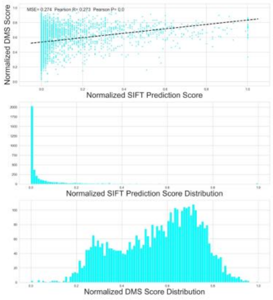

(b)

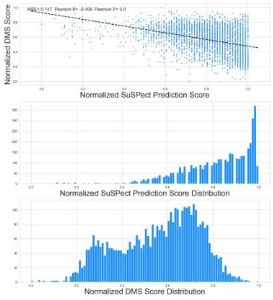

(c)

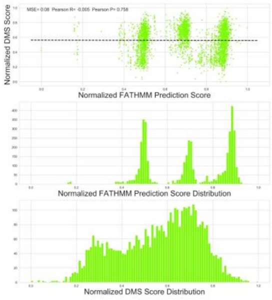

(d)

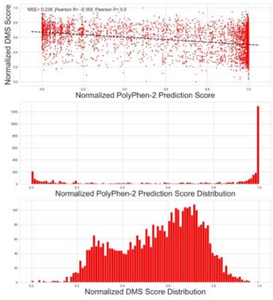

(e)

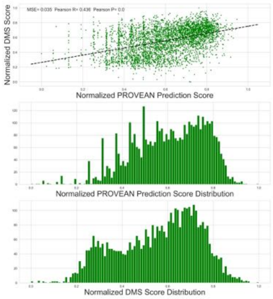

(f)

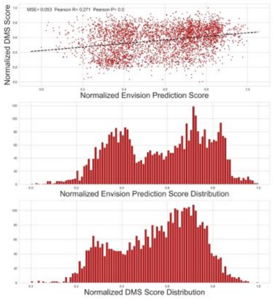

(g)

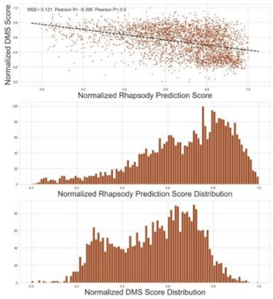

(h)

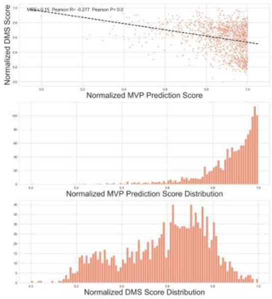

(i)

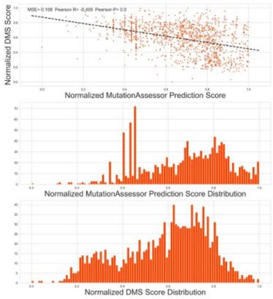

(j)

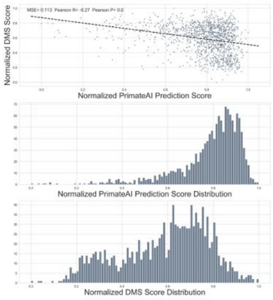

(k)

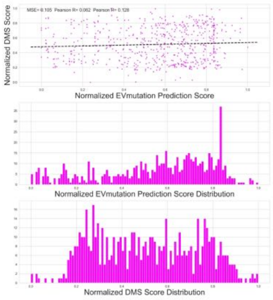

(l)

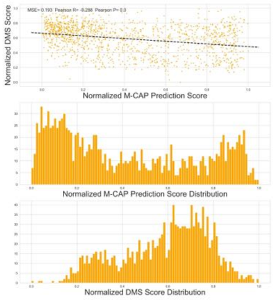

(m)

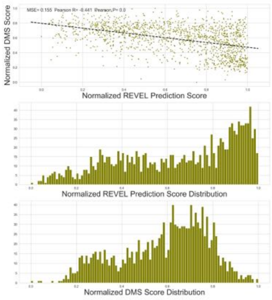

(n)

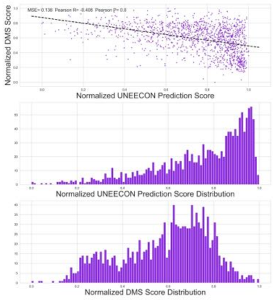

(o)

Figure S21. Linear Regression Between Normalized pten(a) Experimental Scores and Normalized Variant Effect Predictors' Prediction Scores. Values on both axes range from 0 to 1. Dashed black lines give linear least-squared regressions. Distributions of experimental and predicted scores were also plotted within the same subfigure.

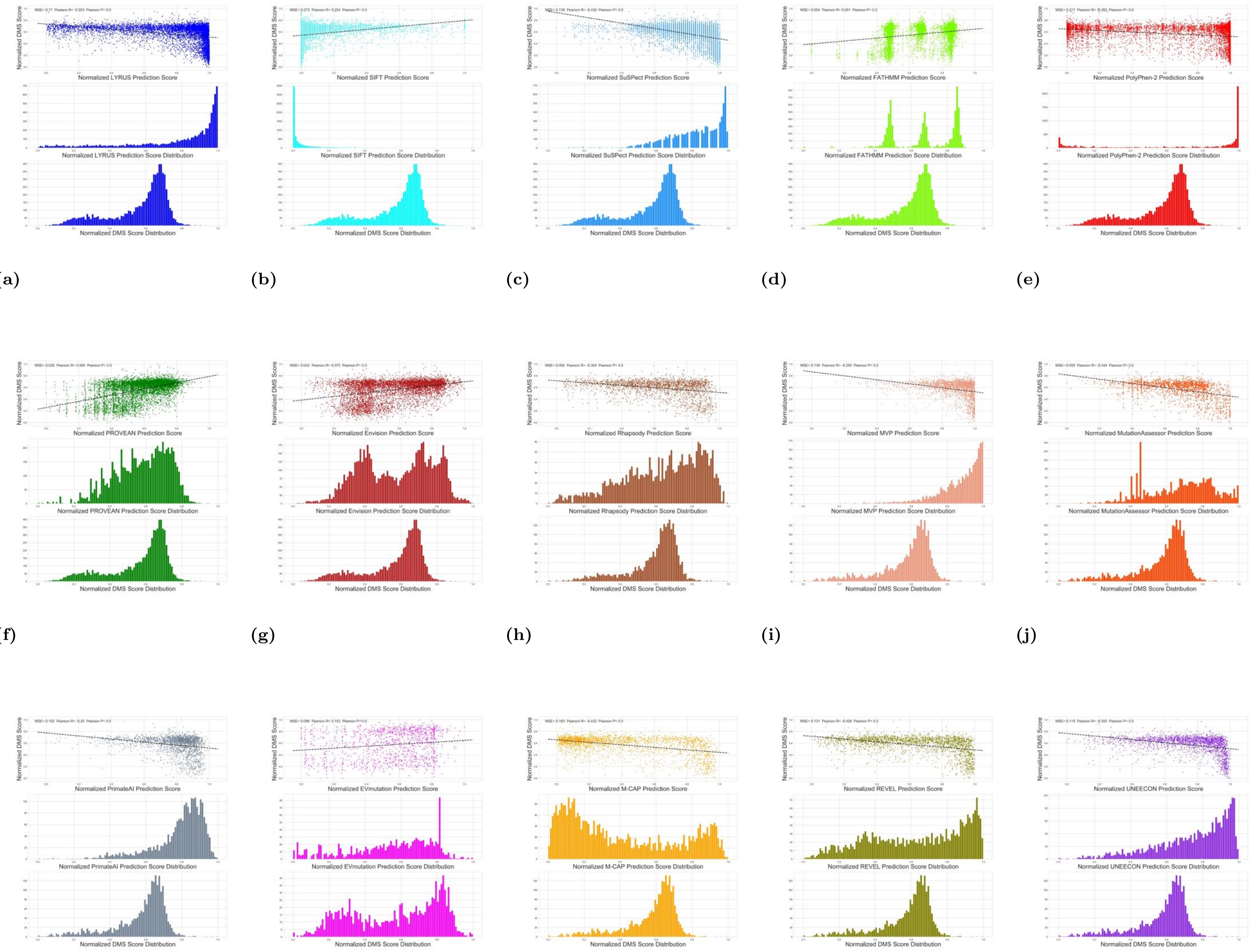

(a) (b) (c) (d) (e)

(f) (g) (h) (i) (j)

(k) (l) (m) (n) (o)

Figure S22. Linear Regression Between Normalized pten(b) Experimental Scores and Normalized Variant Effect Predictors' Prediction Scores. Values on both axes range from 0 to 1. Dashed black lines give linear least-squared regressions. Distributions of experimental and predicted scores were also plotted within the same subfigure.

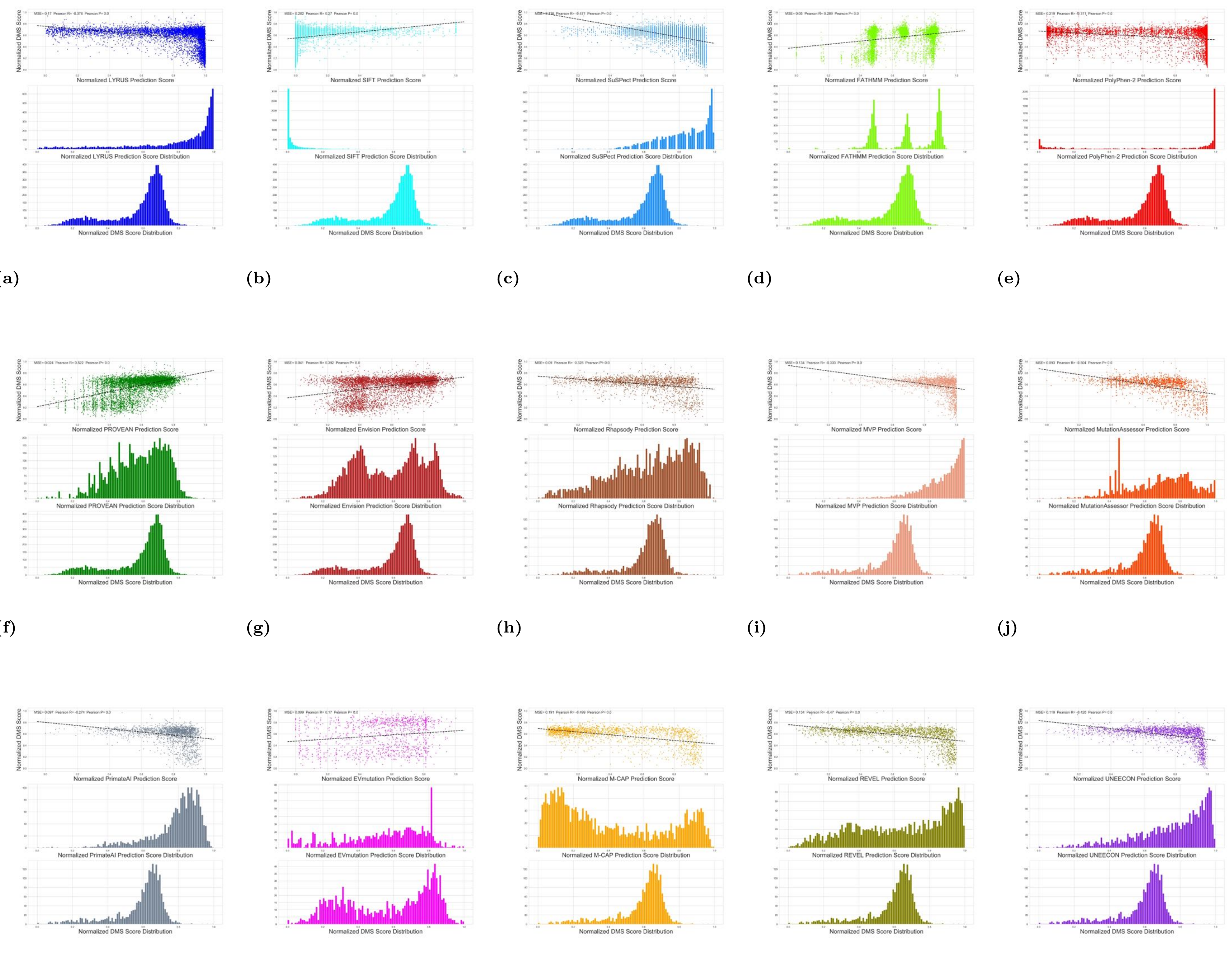

(a) (b) (c) (d) (e)

(f) (g) (h) (i) (j)

(k) (l) (m) (n) (o)

Figure S23. Linear Regression Between Normalized pten(highqual_b) Experimental Scores and Normalized Variant Effect Predictors' Prediction Scores. Values on both axes range from 0 to 1. Dashed black lines give linear least-squared regressions. Distributions of experimental and predicted scores were also plotted within the same subfigure.

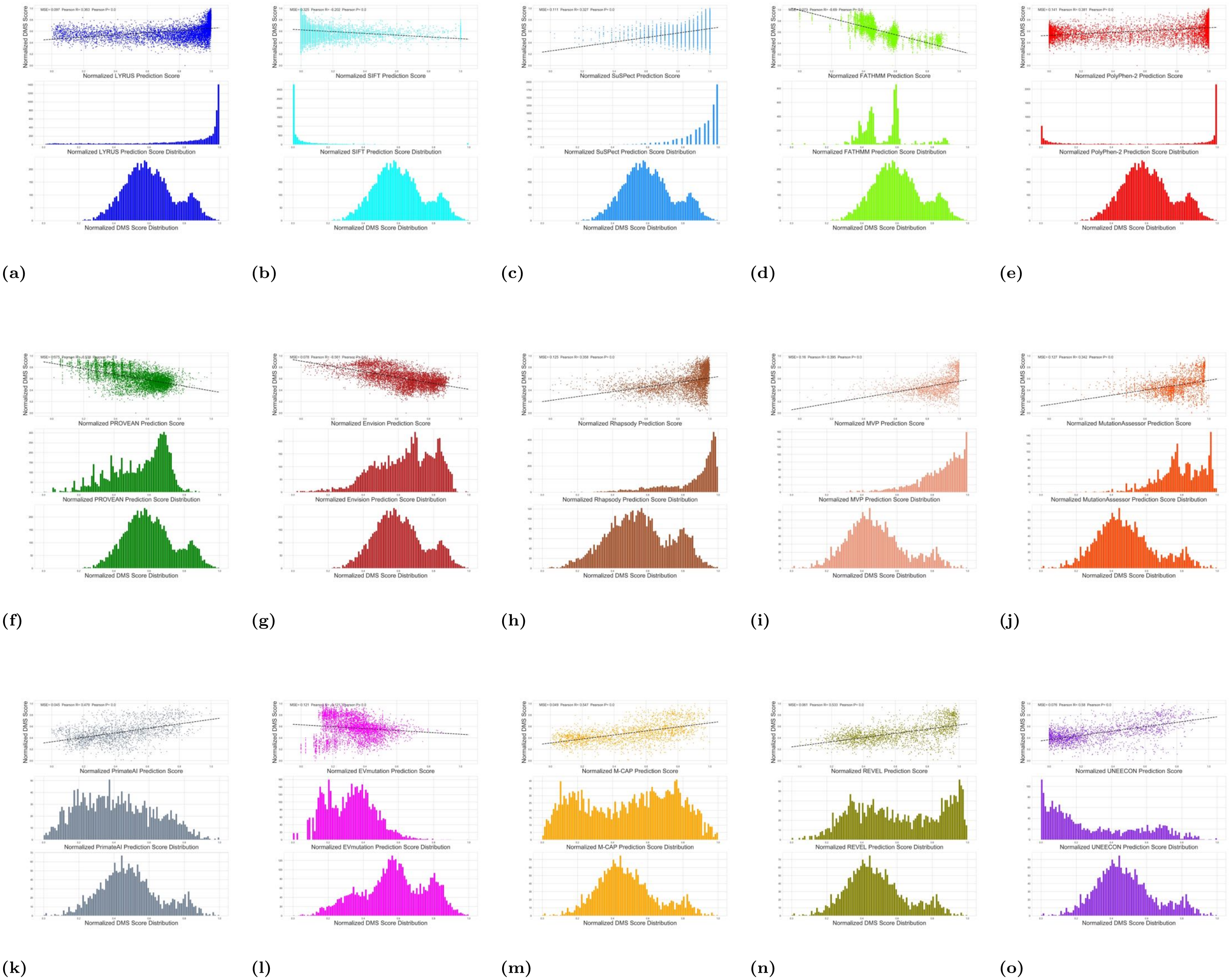

Figure S24. Linear Regression Between Normalized p53(wt_nutlin) Experimental Scores and Normalized Variant Effect Predictors' Prediction Scores. Values on both axes range from 0 to 1. Dashed black lines give linear least-squared regressions. Distributions of experimental and predicted scores were also plotted within the same subfigure.

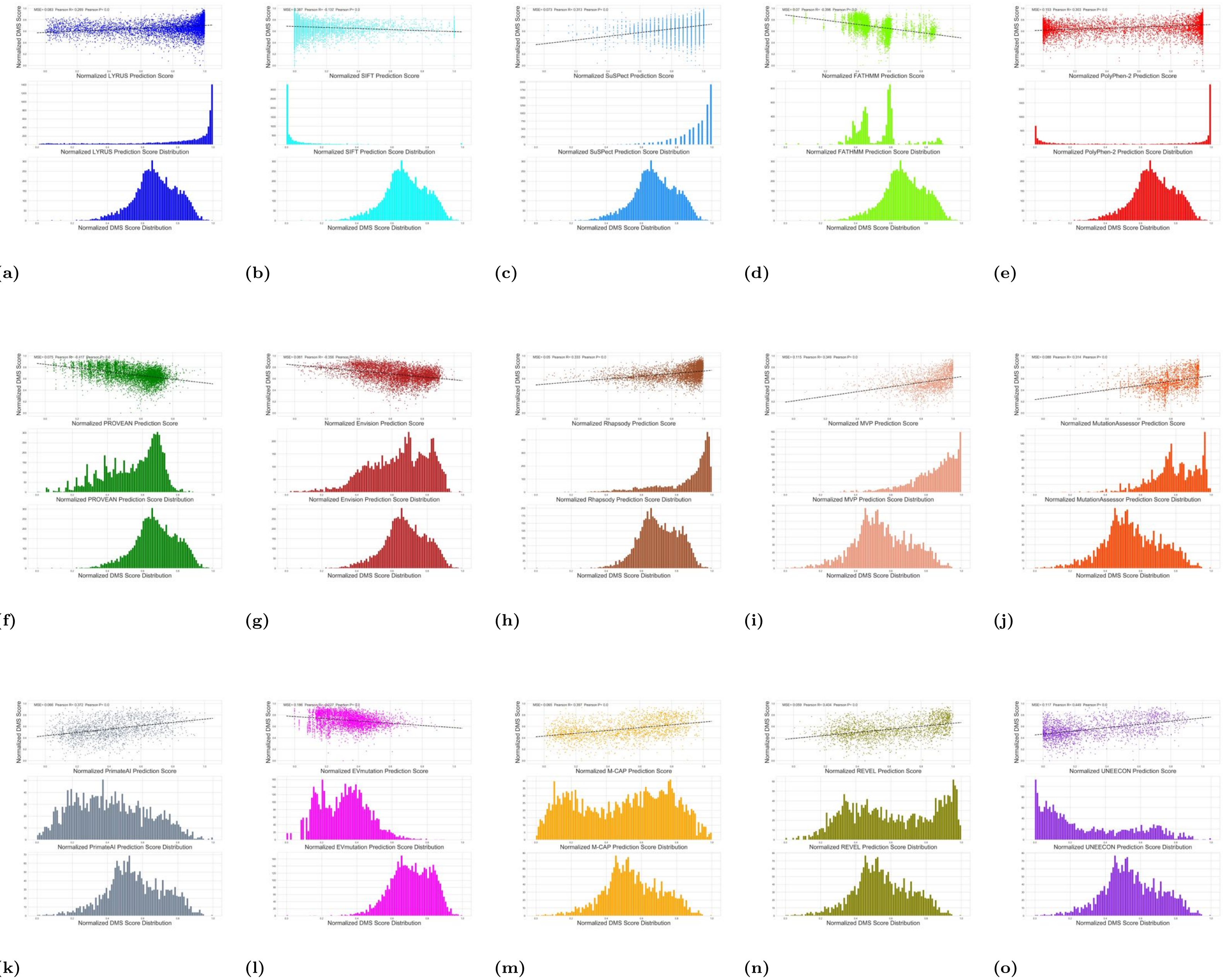

Figure S25. Linear Regression Between Normalized p53(null_nutlin) Experimental Scores and Normalized Variant Effect Predictors' Prediction Scores. Values on both axes range from 0 to 1. Dashed black lines give linear least-squared regressions. Distributions of experimental and predicted scores were also plotted within the same subfigure.

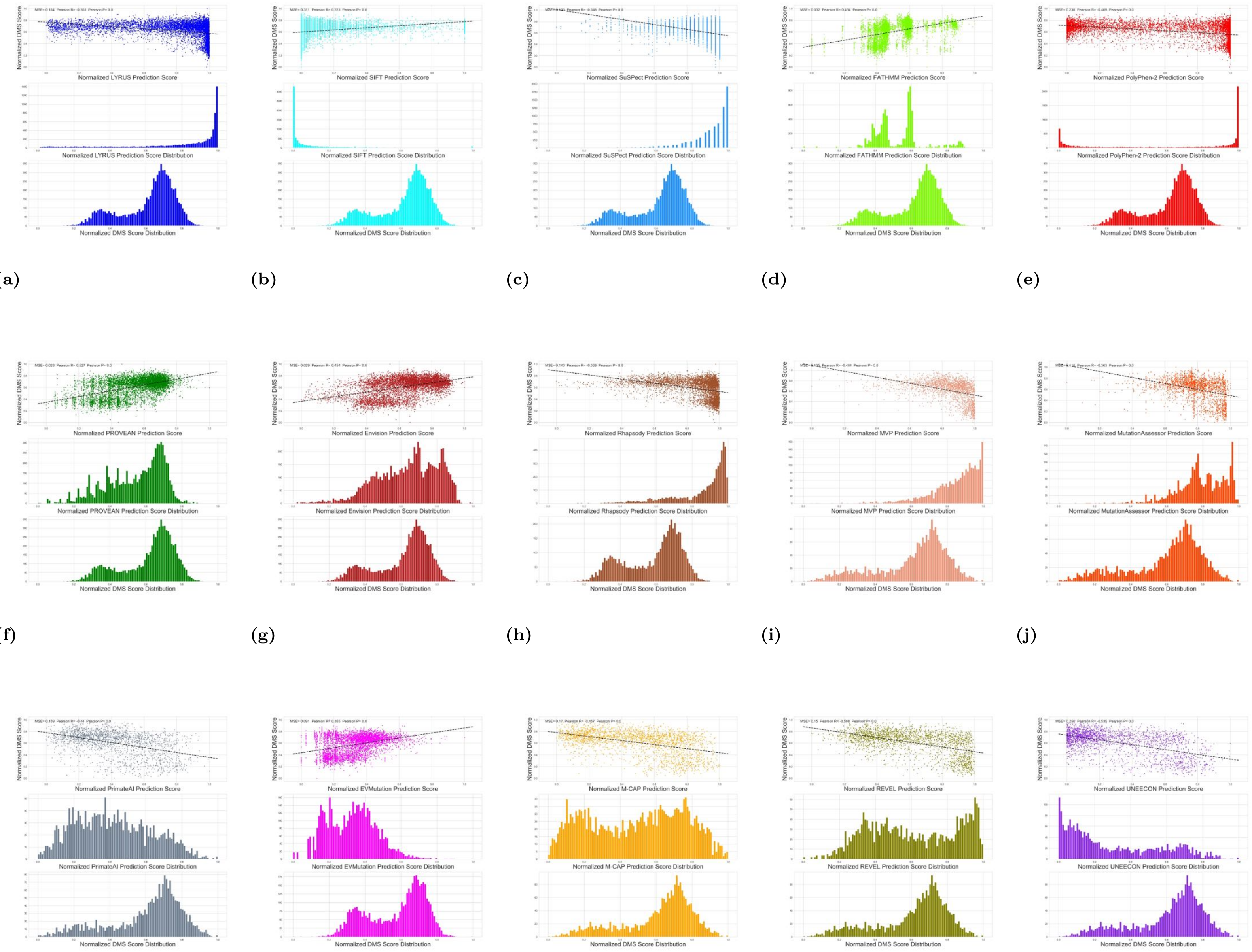

(k) (l) (m) (n) (o)

Figure S26. Linear Regression Between Normalized p53(null_etoposide) Experimental Scores and Normalized Variant Effect Predictors' Prediction Scores. Values on both axes range from 0 to 1. Dashed black lines give linear least-squared regressions. Distributions of experimental and predicted scores were also plotted within the same subfigure.
